# Socio-economic value of data-driven eruption forecasts to balance false alarms against catastrophic loss

**DOI:** 10.1038/s41467-026-77242-4

**Published:** 2026-08-26

**Authors:** Alberto Ardid, David Dempsey, Garry McDonald, Shane Cronin

**Affiliations:** 1 University of Canterbury, Christchurch, New Zealand; 2 Market Economics, Auckland, New Zealand; 3 University of Auckland, Auckland, New Zealand

**Keywords:** Volcanology, Natural hazards, Socioeconomic scenarios, Geography

## Abstract

The adoption of new forecasting methods for volcanic eruptions typically emphasizes predictive accuracy, often overlooking their potential to reduce overall life and economic losses. We introduce a Potential Economic Value (PEV) framework that evaluates forecast utility by balancing precautionary actions against avoidable catastrophic losses (such as mass casualties). Using machine-learning forecasts from continuous seismic data at five volcanoes, we show that non-forecasted eruptions (missed) have disproportionate consequences, compared to false alarms, which generate recurring and manageable disruption. Retrospective analyses of the 2019 Whakaari (New Zealand) and 2014 Ontake (Japan) eruptions, along with three additional volcanoes, indicate that losses could have been reduced by 30–90%, despite generating numerous false alarms. Across all case studies, effective forecasting prioritizes reducing missed eruptions over maximizing accuracy. This supports the use of more precautionary warning thresholds, provided that the resulting increase in alert frequency is managed in ways that maintain public compliance and trust.

## Abstract (web summary)

Volcanic disasters cause widespread economic damage. Here, the authors examine the potential economic value of precautionary action compared to losses finding that losses could be reduced 30-90% even with false alarms.

## Introduction

Volcanic eruptions are among the most destructive natural hazards on Earth, capable of causing widespread disruption, loss of life, and long-term socioeconomic impacts. Globally, 50–70 eruptions occur each year, with a minority causing disproportionate losses^1^. Recent events span a wide spectrum of local to global consequences: the 2010 Merapi eruption in Indonesia killed more than 350 people and forced the evacuation of 400,000, many of whom were permanently displaced^2^. Other large eruptions such as Eyjafjallajökull in 2010, Puyehue–Cordón Caulle in 2011, Calbuco in 2015, La Palma in 2021, and Hunga Tonga–Hunga Ha’apai in 2022, primarily produced widespread infrastructure damage, aviation disruption, and economic losses, with the latter also causing seven fatalities and many injuries^3–9^. Smaller eruptions can also produce severe human impacts despite limited physical damage. The Ontake 2014 eruption (63 fatalities^10^) and the 2019 Whakaari eruption (22 fatalities and dozens of severe injuries^11^) exemplify how short-warning, exposure-driven events can be disproportionately deadly, raising the question of where the true value of forecasting systems lies. If most infrastructure damage, aviation disruption, and economic losses are largely unavoidable, does the primary value of forecasting lie in preventing loss of life?

Despite extended advances in monitoring over the last decades, anticipating eruptions remains challenging. Seismic, geodetic, and gas precursors often herald unrest^12–14^, yet forecasts remain uncertain because genuine precursor signals vary across volcanoes and are difficult to identify and evaluate robustly across eruptive and non-eruptive periods, particularly under out-of-sample conditions^15–17^. Also, some eruptions occur with subtle or absent warnings (Ontake 2014^10^; Whakaari 2016, 2019^11,18^), and most volcanoes lack sufficient recorded events to identify reliable patterns^19^.

Recent data-driven approaches have improved the detection of subtle patterns in monitoring data, producing likelihood of eruption forecasts that can be evaluated under retrospective conditions^20–24^. While some models show encouraging skill, most studies focus on pre-eruptive intervals without comparing against non-eruptive repose^17,25,26^. Plus, objective metrics that account for missed eruptions and false alarms over long periods, such as ROC curves and AUC scores, are necessary to evaluate forecast reliability^19,27–29^.

Alongside accuracy, it is important to consider the socio-economic costs and benefits of forecasting systems. False alarms may trigger unnecessary evacuations, closures of businesses and transport networks, legal disputes, and erosion of public trust^30,31^. Equally, missed eruptions for which evacuation would have been feasible incur financial costs associated with loss of life, injury, and recovery. Hence, forecast value can be framed more generally than predictive skill alone, extended to include its financial performance through mitigated socioeconomic costs. In this study, we address this gap by introducing a quantitative framework that explicitly balances the short-term costs of precautionary action against the avoided losses from catastrophic events. We restrict our attention to costs arising from short-term economic disruption and associated loss-modeling components only, excluding longer-term equilibrium effects^32–35^.

Evaluating a forecast’s socioeconomic value requires estimating costs that are inherently uncertain, either because they are difficult to measure or they vary considerably with context. International benchmarks for human life valuation vary widely (e.g., New Zealand: USD 2.7M^36^; United States: USD 12.5 M per fatality^37^), and communities differ in acceptable disruption levels and risk tolerance^15,38,39^. Both false alarms and missed eruptions carry psychological costs. Both can result in a loss of institutional trust, while repeated alerts may erode compliance^40,41^ and missed events cause trauma^31,41^. However, the consequences can be asymmetric: false alarms generate recurring but manageable costs, whereas missed eruptions can lead to substantial loss of life^42,43^. Despite the clear need for eruption forecast evaluation that incorporates socio-economic consequences, no standardized framework exists for comparing forecast value across volcanoes with differing hazard profiles and exposure contexts.

Here, we introduce the Potential Economic Value (PEV) framework to volcanology as a generalizable and interpretable metric, where generalizability refers to the ability to apply the same dimensionless cost–loss formulation across different volcanoes. Originally developed for meteorology and aviation^44,45^ and now applied in flood, drought, and wildfire risk management^46–49^, PEV balances the short-term costs of precautionary actions (false alarms) against the avoided losses when forecasts correctly anticipate eruptions, yielding a single dimensionless measure of forecast utility. We demonstrate the PEV framework on multi-year eruption forecasts derived from a machine-learning model^19^ at five volcanoes that collectively span a spectrum of eruption frequencies, monitoring histories, and socio-economic settings. Two cases, Whakaari (New Zealand) and Ontake (Japan), provide documented human and economic losses, enabling retrospective evaluation of the framework. Three additional applications (Ruapehu (New Zealand), Copahue (Chile/Argentine), Bezymianny (Russia)) serve as stress tests, illustrating PEV’s generalizability to hypothetical high-impact scenarios and data-limited systems.

## Results

### Framework evaluation: retrospective cases at Whakaari and Ontake

We begin with two cases where documented casualties and economic losses enable retrospective evaluation of the PEV framework.

**Fig. 1:**
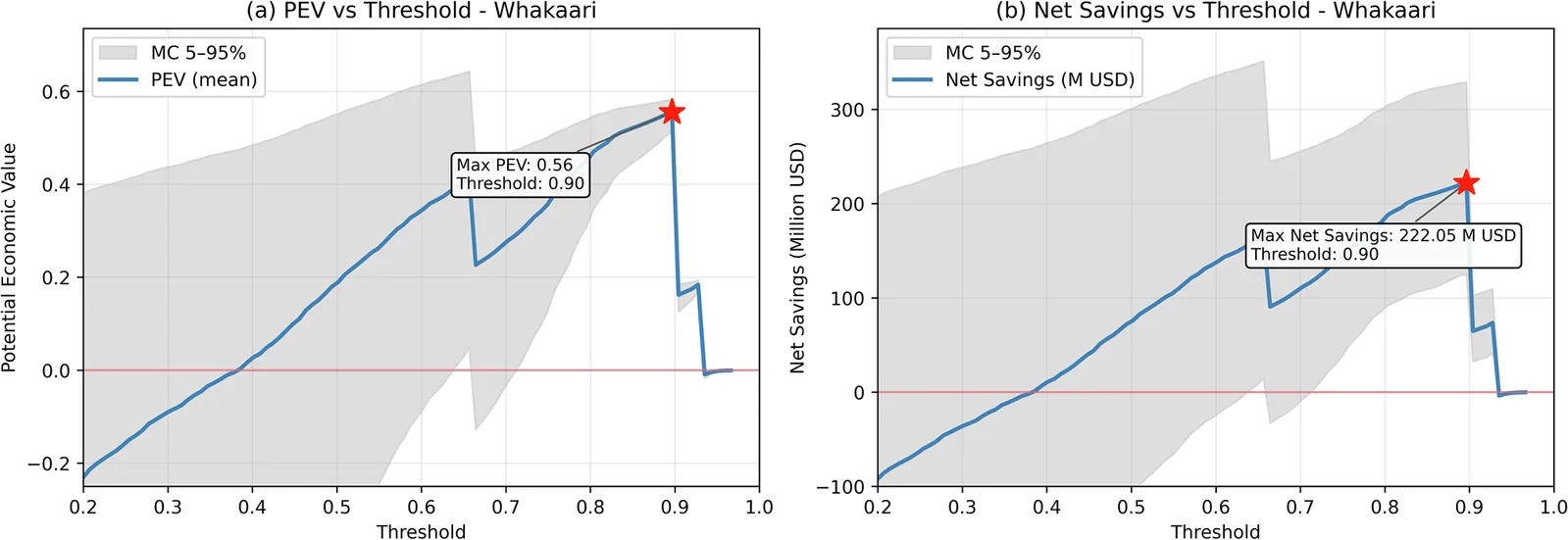
Threshold optimization for Whakaari volcano eruption forecasting using tailored machine learning models. **a** Potential Economic Value (PEV) versus forecast threshold, showing the trade-off between detection sensitivity and economic efficiency. The gray shaded area represents Monte Carlo uncertainty (5th-95th percentile) around the mean PEV. **b** Net economic savings versus threshold, quantifying the monetary benefits of forecasting in millions of USD. Both metrics peak at threshold τ = 0.89, indicating optimal forecast sensitivity that balances early warning benefits against false alarm costs.

For the forecast model applied to Whakaari^19^, forecast value (PEV) is maximized at intermediate decision thresholds that balance false alarms against missed eruptions (Fig. 1). At low thresholds (τ < 0.5), frequent false alarms dominate expected costs, whereas at high thresholds (τ > 0.9), missed eruptions increasingly drive losses, causing PEV to decline. This trade-off produces a characteristic hump-shaped PEV curve (Fig. 1a), a well-established feature of cost–loss decision systems^45^. Monte Carlo ensemble sampling suggests that while absolute PEV values shift under different cost assumptions, the location of the optimum remains relatively stable near τ ≈ 0.85–0.90. At this threshold, forecasts avoid up to ~USD 220 M in losses over the forecast period (Fig. 1b).

In operational terms, the decision threshold τ is the forecast eruption probability above which protective action is initiated: when the model output exceeds τ, the framework recommends precautionary measures such as access restrictions, evacuation of exposed populations, or heightened monitoring posture. A lower τ produces more alerts (including more false alarms), while a higher τ produces fewer alerts but risks missing eruptions preceded by weaker precursory signals.

**Fig. 2:**
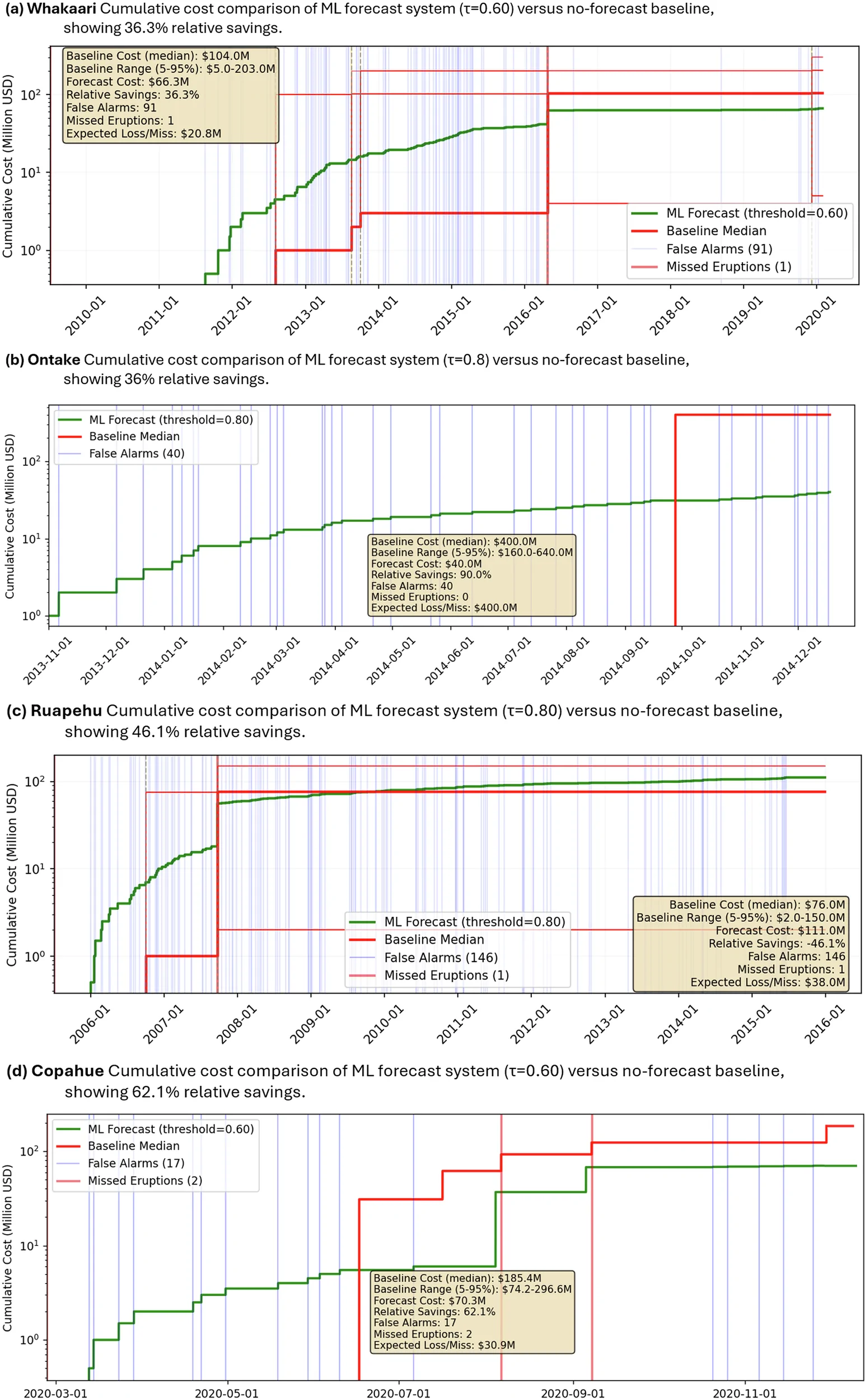
Cumulative cost comparison of ML forecast systems versus no-forecast baselines for Whakaari, Ontake, Ruapehu and Copahue volcanoes. Green lines show cumulative costs with ML forecasts at optimal thresholds; red lines show baseline median costs (no forecast). Vertical markers indicate false alarms (blue) and missed eruptions (red dashed). Summary statistics in each panel quantify economic performance: **a** Whakaari (2010–2020, τ = 0.60) achieved 36.3% relative savings (tested in 5 recorded eruption); **b** Ontake (2013-2014, τ = 0.80) achieved 90.0% relative savings with zero missed eruptions (only tested in one recorded eruption); **c** Ruapehu (2006–2016, τ = 0.80) resulted in 46.1% relative savings due to frequent false alarms (tested in two recorded eruption). **d** Copahue (during 2020, τ = 0.60) resulted in 40.1% relative savings due to frequent false alarms (tested in five recorded eruption).

A retrospective analysis of a 12-year monitoring period at Whakaari shows that the particular forecast evaluated here could have reduced cumulative costs by >30% relative to a no-forecasting baseline in which protective actions are never taken (Fig. 2a). This result is obtained using a decision threshold of τ = 0.6, which is lower than the value-maximizing threshold identified in the preceding PEV analysis (τ ≈ 0.85–0.90), and therefore represents a deliberately precautionary choice that favors reducing missed eruptions at the expense of more frequent false alarms. As a result, the reported cost reduction should be interpreted as conservative; greater savings would have been achievable had the optimal threshold been known in advance.

For the selected cost-modeling assumptions (20% of missed eruptions cause catastrophic losses of USD 20 M; each false alarm costs USD 0.5 M; see Methods and Supplementary Text S5), total costs accumulate in small increments from repeated false alarms, whereas the no-forecast baseline exhibits large jumps whenever an eruption is missed. In this case, frequent minor costs are less damaging than rare catastrophic outcomes. Short-term forecast costs can occasionally exceed the no-forecast baseline, because only a subset of missed eruptions are modeled to incur catastrophic losses; consequently, accepting a high false-alarm rate is economically rational only over sufficiently long-time horizons.

**Fig. 3:**
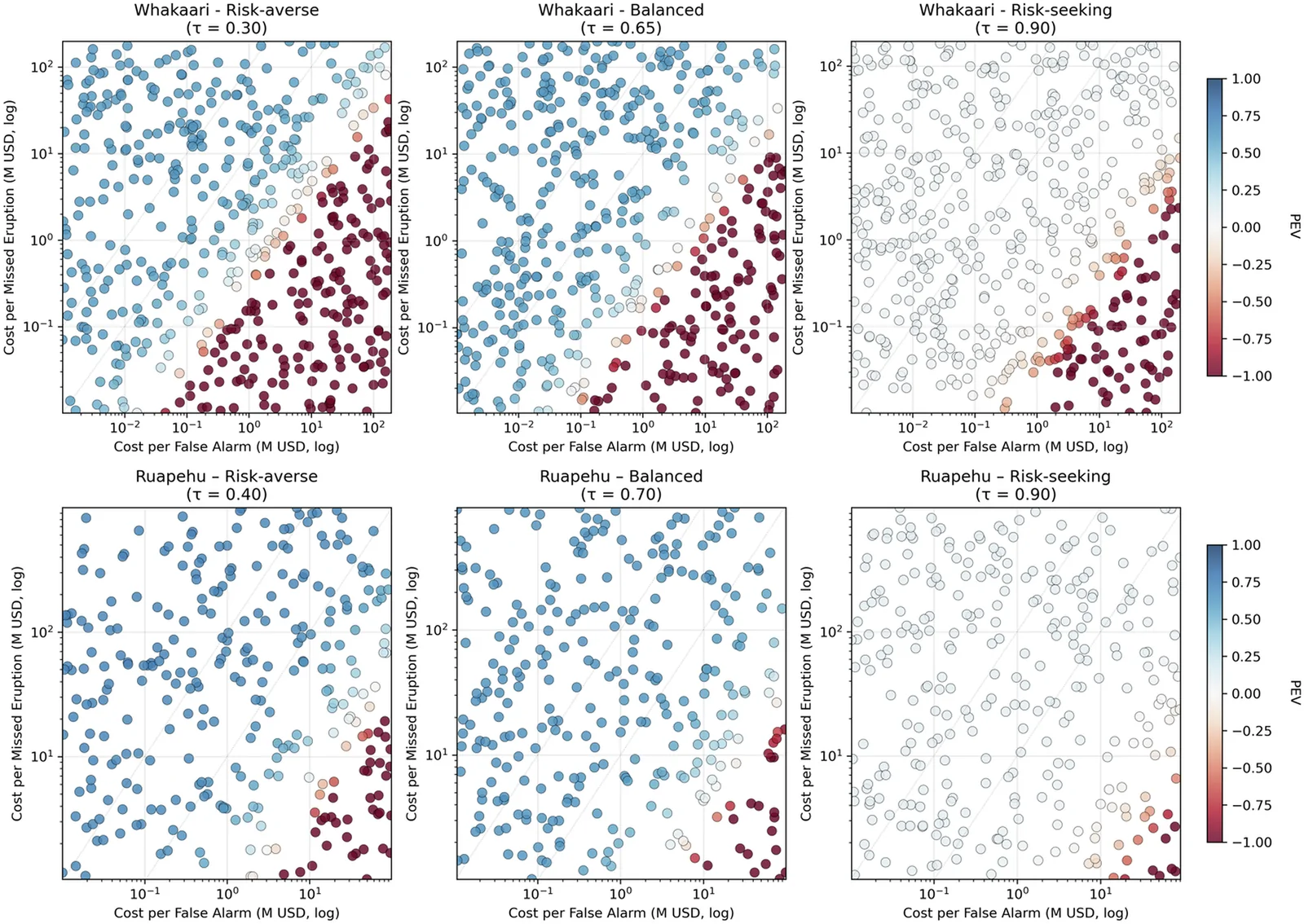
Sensitivity of potential economic value (PEV) to cost parameters for Whakaari (top row) and Ruapehu (bottom row). Each panel shows how PEV varies across false-alarm costs (C) and missed-eruption losses (L) for three forecast thresholds—risk-seeking (τ = 0.30/0.40), balanced (τ = 0.65/0.70), and risk-averse (τ = 0.90). Each point represents a unique C–L combination, colored by PEV (blue = economic loss; red = economic benefit). The diagonal PEV = 0 boundary illustrates the trade-off between false-alarm and missed-eruption costs: lower thresholds favor detection (positive PEV when L ≫ C), while higher thresholds require very low false-alarm costs to remain beneficial. The geometry of these plots highlights that balanced thresholds provide the broadest region of robust, positive value under uncertainty, particularly in speculative scenarios such as Ruapehu.

We examined PEV robustness by varying false-alarm costs (C) and missed-eruption losses (L) across several orders of magnitude (Fig. 3). Across plausible cost ratios, PEV remains positive, indicating that forecasts reduce expected losses even when valuations are uncertain. Threshold selection shapes this robustness: risk-seeking thresholds (τ = 0.30) yield positive value only when L ≫ C, whereas balanced thresholds (τ ≈ 0.65) expand the positive-PEV region substantially. Risk-averse thresholds (τ ≈ 0.90) erode value rapidly, as even small numbers of missed eruptions collapse economic benefit.

Ontake provides a complementary test of the PEV framework to Whakaari, representing a high-exposure, land-based volcanic setting with public access, minimal structural damage, and dominant life-safety consequences. Using documented casualties and exposure at Ontake, a retrospective scenario analysis suggests that, under assumptions of timely precautionary action, a short-term forecast could have substantially reduced exposure-related losses while requiring repeated precautionary alerts over the analysis period (Fig. 2b).

If there was a forecasting method available at the time, and it was acted upon with evacuation planning, retrospective analysis shows that a forecast at Ontake could have reduced human and exposure-related losses by ~90%, avoiding short-term impacts despite 40 false alarms (Fig. 2b). This is consistent with the findings for Whakaari: when catastrophic human losses dominate the cost structure, even imperfect forecasts with high false-alarm rates provide substantial value over the long term.

Unlike Whakaari, where forecast actions primarily affect commercial operators and tourism access, Ontake illustrates a setting where precautionary actions are limited to temporary closure of public trails and summit access. As a result, false alarm costs are relatively modest compared to the potential consequences of a missed eruption, which are dominated by life-safety losses. This contrast shows how PEV responds not only to forecast performance, but also to differences in exposure structure and institutional control.

Ontake represents a challenging test case for forecast-based decision-making, as the retrospective analysis involves a large number of precautionary alerts relative to a single catastrophic outcome. It is important to note, however, that such high false-alarm rates are not an inherent feature of data-driven eruption forecasting. At volcanoes with more persistent and coherent precursory signals, short-term forecasting models can generate substantially fewer alerts while still providing clear advance warning. For example, out-of-sample application of a generalized forecasting model at Mount St. Helens showed elevated eruption likelihoods confined to a narrow window immediately preceding eruption, with limited spurious excursions during non-eruptive periods^19^. This contrast suggests that Ontake is a conservative stress test of the PEV framework, rather than a typical operational scenario.

### Framework generalizability: hypothetical high-impact scenarios

Having examined retrospective cases, we tested the generalizability of PEV to volcanoes lacking recent major loss events but facing credible high-impact scenarios.

In high-exposure alpine settings like Mount Ruapehu, where lahars threaten people working in and visiting ski fields, accommodation areas, and hiking trails, short-term forecasts could reduce expected losses by enabling temporary access restrictions and evacuation of people from exposed areas (Fig. 2c).

At Ruapehu, false alarm costs accumulate steadily, but in scenario-based analysis the avoidance of rare catastrophic impacts dominates net expected value. Although only two minor eruptions occurred during the 14-year observation period, and neither resulted in casualties, this outcome reflects favorable timing and limited exposure rather than the absence of credible life-safety risk. Under plausible assumptions about the probability that a missed eruption coincides with high exposure (e.g., during ski season or peak visitation), ensemble modeling indicates that precautionary access restrictions could reduce expected losses by >40%. Toward the end of the observation period, forecast costs exceed the no-action baseline, reflecting an extended period without a high-impact event; however, this behavior is an expected feature of systems in which impact is realized intermittently, and highlights that apparent short-term ‘success’ without precautionary action can represent an extended period of risk accumulation rather than safety.

Sensitivity analysis (Fig. 3) reveals that balanced thresholds (τ ≈ 0.70) maintain positive PEV across wide cost ranges, whereas risk-averse thresholds (τ = 0.90) eliminate value in the absence of recent catastrophic losses. This illustrates a practical challenge: in the absence of recent damaging eruptions to calibrate perceived risk, high alert thresholds may seem cautious, yet they increase expected losses by allowing rare but catastrophic events to be missed.

Applications to Copahue and Bezymianny (Table 1) further demonstrate framework generalizability across volcanic systems with contrasting dominant avoidable loss pathways. At Bezymianny, short-term forecast value is driven primarily by aviation risk mitigation, as explosive eruptions frequently generate ash clouds that intersect major trans-Pacific air routes, while ground-based human exposure is minimal due to the volcano’s remote location and restricted access^50^. In this context, precautionary actions consist mainly of airspace management and flight rerouting rather than evacuation of exposed populations.

**Table 1:** Performance comparison of tailored, generalized, and RSAM forecasting models for Whakaari, Bezymianny, and Copahue volcanoes

Volcano	Model	N Eruptions	Threshold	AUC	PEV	Savings (M USD)
**Whakaari**	tailored	5	0.89	0.88	0.55	219.98
**Whakaari**	generalized	5	0.62	0.96	0.75	300.43
**Whakaari**	rsam	5	94	0.85	0.47	186.93
**Bezymianny**	tailored	5	0.86	0.76	0.35	265.45
**Bezymianny**	generalized	5	0.88	0.69	0.38	284.74
**Bezymianny**	rsam	5	94	0.7	0.38	283.69
**Copahue**	tailored	6	0.59	0.78	0.39	162.14
**Copahue**	generalized	6	0.62	0.88	0.45	189.42
**Copahue**	rsam	6	87	0.76	0.37	154.75

Generalized models demonstrate consistent cross-volcano value, achieving the highest economic savings (189–300 M USD) and AUC values (predictive accuracy and economic performance) across all three volcanoes despite being trained on data from other volcanic systems. Values represent net cumulative savings over the monitoring periods analyzed (Whakaari: 2010–2020; Bezymianny: 2007–2009; Copahue: 2019–2020) at optimal thresholds (τ ≈ 0.7–0.9). This highlights the transferability and economic benefits of generalized eruption forecasting approaches.

In contrast, Copahue represents a system where forecast value is dominated by evacuation and access-control logistics. Eruptive activity necessitates the evacuation of nearby communities and large-scale movement of people, livestock, and infrastructure across the Chile–Argentina border, generating short-term economic and operational costs that are sensitive to alert timing^51^. While Copahue also produces ash affecting aviation, its principal avoidable losses arise from ground-based exposure and evacuation management.

Accordingly, at Copahue we explored the sensitivity of forecast value to alert threshold choice by quantifying how false alarms, missed eruptions, and economic outcomes vary across τ under fixed cost–loss assumptions. This analysis shows that positive forecast value is not confined to a single optimal threshold, but arises across a range of intermediate thresholds where reducing the likelihood of rare, high-impact misses outweighs the cumulative cost of precautionary actions (Supplementary Text S1; Figs. S1–S2).

### Comparison of PEV across forecasting models

To assess how PEV depends on forecast model, we evaluated the metric on three different models: (1) a machine-learning model tailored to a specific volcano^11,52^, (2) a model that uses transfer-learning to pool precursor information from multiple volcanoes^19^, and (3) a benchmark forecast based on the unmodified seismic data (RSAM) that these machine-learning models learn from. As tailored models can only be developed for a small subset of volcanoes with sufficient recorded eruptions, we have restricted this analysis to Whakaari, Bezymianny and Copahue.

For Whakaari, the generalized model yielded highest PEV (0.38–0.75) and greatest cost savings, with the tailored model performing slightly worse (Table 1). RSAM produced lower but still positive value, demonstrating that even unmodified amplitude metrics have forecasting and decision-making utility. The same comparison for Copahue and Bezymianny also indicates that generalized models achieve the highest positive economic value (189–300 M USD savings), despite training on data from other volcanic systems (Table 1). This suggests that models incorporating generalized multi-volcano precursory signals^19^ offer operational advantages for data-limited settings where local eruption histories are insufficient.

An important operational consideration is the trade-off between forecast performance and interpretability. RSAM, as a simple seismic amplitude metric, is straightforward to implement, explain to stakeholders, and audit, and our analysis confirms that it provides positive economic value at all tested volcanoes (Table 1). The ML models provide additional value (USD 30–115 M in marginal savings over RSAM across the tested volcanoes) by detecting subtler precursory patterns, and are partially interpretable through feature importances and physically meaningful input features^19,21^. In operational settings, RSAM and ML models need not be competing alternatives: RSAM can serve as a transparent baseline that agencies and the public can readily understand, while ML outputs provide supplementary decision support with quantifiable additional value^53^. The choice of which to deploy depends on local institutional capacity, data infrastructure, and the communication context in which forecasts are used.

The finding that generalized models outperform tailored models (Table 1) is consistent with a well-established regularity in multi-task machine learning applied to data-scarce settings. In hydrological prediction^54^, it has been shown that a single model trained across 531 catchments outperformed individually calibrated models at each site, because the pooled network captures process invariances that small per-site samples cannot resolve. With 5–6 eruptions per volcano (Table 1), our dataset is in the regime where pooled training has been shown to outperform per-site calibration in other domains. This result also supports the interpretation that seismic precursory features are more universal across volcanic systems than previously assumed, consistent with the transfer-learning findings of Ardid and Dempsey et al., 2025^19^.

## Discussion

### Advantages of the PEV framework in volcanology

Application of the PEV framework to Whakaari and Ontake suggests that a substantial fraction (30–90%) of preventable losses in these retrospective case studies could, in principle, be reduced through timely precautionary action. A key insight from these analyses is that forecast utility derives primarily from reducing the probability of rare, high-consequence outcomes rather than from maximizing conventional accuracy metrics alone. This behavior is consistent with established cost–loss theory in meteorology^45^, but has not been routinely incorporated into eruption forecasting evaluation. By focusing on avoidable losses driven by exposure rather than eruptive magnitude, the PEV framework aligns with evidence that vulnerability dominates eruption impact across diverse volcanic settings (see Supplementary Text S2).

An additional strength of PEV is that it provides a common, dimensionless structure for evaluating forecast value across volcanic systems with differing eruption frequencies, exposure patterns, and cost structures. By focusing on relative trade-offs between false alarms and missed events, rather than absolute costs, the framework remains informative even when economic valuations are uncertain by orders of magnitude.

## Operational challenges

Although the PEV analysis identifies a value-maximizing decision threshold in hindsight, this threshold is not known a priori in operational settings. In practice, agencies must choose alert thresholds under uncertainty, informed by imperfect knowledge of future eruption behaviour, exposure, and societal tolerance for false alarms versus missed events. Importantly, our sensitivity analyses indicate that while absolute economic value varies with cost assumptions, the location of the PEV optimum remains relatively stable across a broad range of plausible cost ratios. This stability suggests that threshold selection can be robust to substantial uncertainty in economic valuation, supporting the use of PEV as a decision-support tool rather than a prescriptive optimization rule.

Applying the PEV framework in operational settings presents important challenges. Most notably, the decision threshold that maximizes expected value can only be identified retrospectively and is therefore not known with foresight. In practice, agencies must select thresholds under uncertainty, informed by imperfect knowledge of future eruption behaviour, exposure patterns, and their tolerance for false alarms relative to missed events. As a result, PEV should be interpreted as a decision-support tool for exploring trade-offs, rather than as a prescriptive rule for optimal action.

A further limitation arises at volcanoes with sparse eruptive records, where prospective estimation of false-alarm rates and missed-eruption probabilities is inherently uncertain. In such settings, forward simulation and sensitivity analysis are essential for examining plausible long-term cost–loss trajectories and for avoiding overconfidence based on limited historical experience.

In practice, we recommend that agencies adopt a robust initial threshold, one that maintains positive PEV across a broad range of plausible cost assumptions, rather than attempting to identify a value-maximizing threshold a priori. Our sensitivity analyses (Fig. 3) indicate that balanced thresholds (τ ≈ 0.65–0.70) sustain positive PEV across wide cost-ratio ranges, making them robust initial choices. This approach parallels earthquake early warning practice, where alert thresholds are set below the target damage threshold to bound miss probability at a policy-specified ceiling, accepting a measurable increase in false alerts^55,56^. As forecast-outcome data accumulate, thresholds can be refined through Bayesian updating of the posterior expected loss, following established methods in flood and weather forecasting^57,58^. This sequential approach protects against the catastrophic-regret scenario in which a too-permissive threshold is chosen and a major eruption is missed.

Finally, the application of PEV requires assigning monetary values to human life and other impacts, a methodologically necessary but ethically constrained practice in risk analysis^59^. Values such as the Value of Statistical Life (VSL) vary substantially across jurisdictions, reflecting differences in regulatory practice, income levels, and policy conventions rather than any normative assessment of individual lives. Moreover, economic valuation cannot fully capture non-monetary consequences such as psychological trauma, long-term displacement, or loss of cultural and social assets^31,41^. For this reason, PEV should not be interpreted as a comprehensive measure of societal impact. Rather, it provides a structured means of comparing alternative warning strategies under uncertainty. Importantly, sensitivity analyses show that the qualitative conclusions of this study are robust across order-of-magnitude variation in cost assumptions, indicating that the potential value of forecasting does not hinge on any single valuation choice. Decisions about acceptable risk and warning practice must therefore be informed by, but not determined solely through, economic optimization, and should be situated within broader deliberative and governance processes^15,38^.

The false-alarm cost C is primarily composed of fixed operational expenditures (evacuation logistics, lost revenue, emergency service mobilization) that are largely independent of VSL, whereas the missed-eruption loss L scales with the chosen VSL benchmark through its fatality component. Increasing VSL therefore decreases the cost-loss ratio C/L^60^ and shifts the optimal threshold toward more precautionary values, consistent with the ethical intuition that greater weight on human life should lower the bar for protective action. The sensitivity analyses in Fig. 3 show that PEV remains positive across a wide range of C/L values, and the broad, flat optimum means that shifts in optimal threshold location across plausible VSL ranges are small. The framework’s policy-relevant conclusions, namely which thresholds provide positive value and that balanced thresholds are preferred, are therefore robust to the choice of VSL benchmark.

### Distribution of forecast-related costs and societal acceptance

Forecast-informed decision-making redistributes the short-term costs and consequences associated with volcanic risk, and these are not borne uniformly across affected groups. For example, at Whakaari, precautionary actions triggered by elevated forecasts primarily impose costs on tourism operators and visitors through disrupted access, lost revenue, and travel interruptions. In contrast, the consequences of missed eruptions are dominated by life-safety impacts borne by exposed individuals (including visitors, workers, and operators) as well as by public agencies responsible for emergency response, recovery, and legal accountability (for an hypothetical analysis on this asymmetry in cost incidence under false-alarm and missed-eruption outcomes, see Supplementary Text S3 and Figure S3).

Within the PEV framework, forecast value arises when frequent, relatively low-impact disruptions are exchanged for a reduced probability of rare, high-consequence outcomes. This exchange highlights a central challenge for operational forecasting: the groups that experience the immediate costs of precautionary actions are not necessarily those most directly affected by avoided catastrophic losses. As a result, societal acceptance of frequent precautionary alerts depends not only on forecast performance, but also on how these uneven cost burdens are perceived, communicated, and managed over time.

We emphasize that PEV is intended as a decision-support tool for facilitating transparent stakeholder deliberation, not as a prescriptive metric that determines policy. A stakeholder-partitioned analysis, decomposing aggregate PEV into private-sector costs (e.g., tourism revenue losses from closures) and public-safety gains (e.g., avoided fatalities and emergency response costs), can inform how the costs and benefits of precautionary action are distributed across affected groups. Supplementary Text S3 provides an illustrative decomposition for Whakaari. Formal methods for such distributional analysis are well-established in environmental regulatory analysis^61^, and represent a natural extension of the PEV framework for site-specific policy application.

### Operational integration and behavioral considerations

Successful implementation of PEV-informed forecasting requires addressing both technical and behavioral dimensions. Research on warning compliance shows that repeated false alarms can erode public response by reducing perceived credibility and willingness to act, a phenomenon commonly referred to as the cry-wolf effect^40,62^, while missed eruptions can cause severe loss of institutional trust and long-lasting societal impacts^63^. Historical volcanic crises illustrate this asymmetry: the 1976–77 La Soufrière evacuation^64^ (Saint Vincent, Caribbean), which was not followed by eruption, was retrospectively judged justified, whereas failures to warn at 1975 Nevado del Ruiz eruption^65^ (Colombia), Ontake (2014), and Whakaari (2019), generated enduring criticism. Importantly, evidence from risk-communication research suggests that transparent communication of uncertainty can sustain credibility even when forecasts are imperfect^66^.

A central behavioral challenge is therefore managing societal response to frequent precautionary warnings. Compliance erodes most strongly when alerts are perceived as binary predictions that repeatedly “fail” to materialize. Addressing this could requires, for instance, reframing alerts as indicators of elevated risk rather than definitive eruption statements. Analogous approaches are widely used in other hazard domains: fire-danger rating systems, for example, routinely signal heightened risk and recommend precautionary behaviour without implying that a wildfire is imminent^67,68^. A similar emphasis on relative risk and proportional action may help improve societal acceptance of precautionary volcanic warnings.

**Fig. 4:**
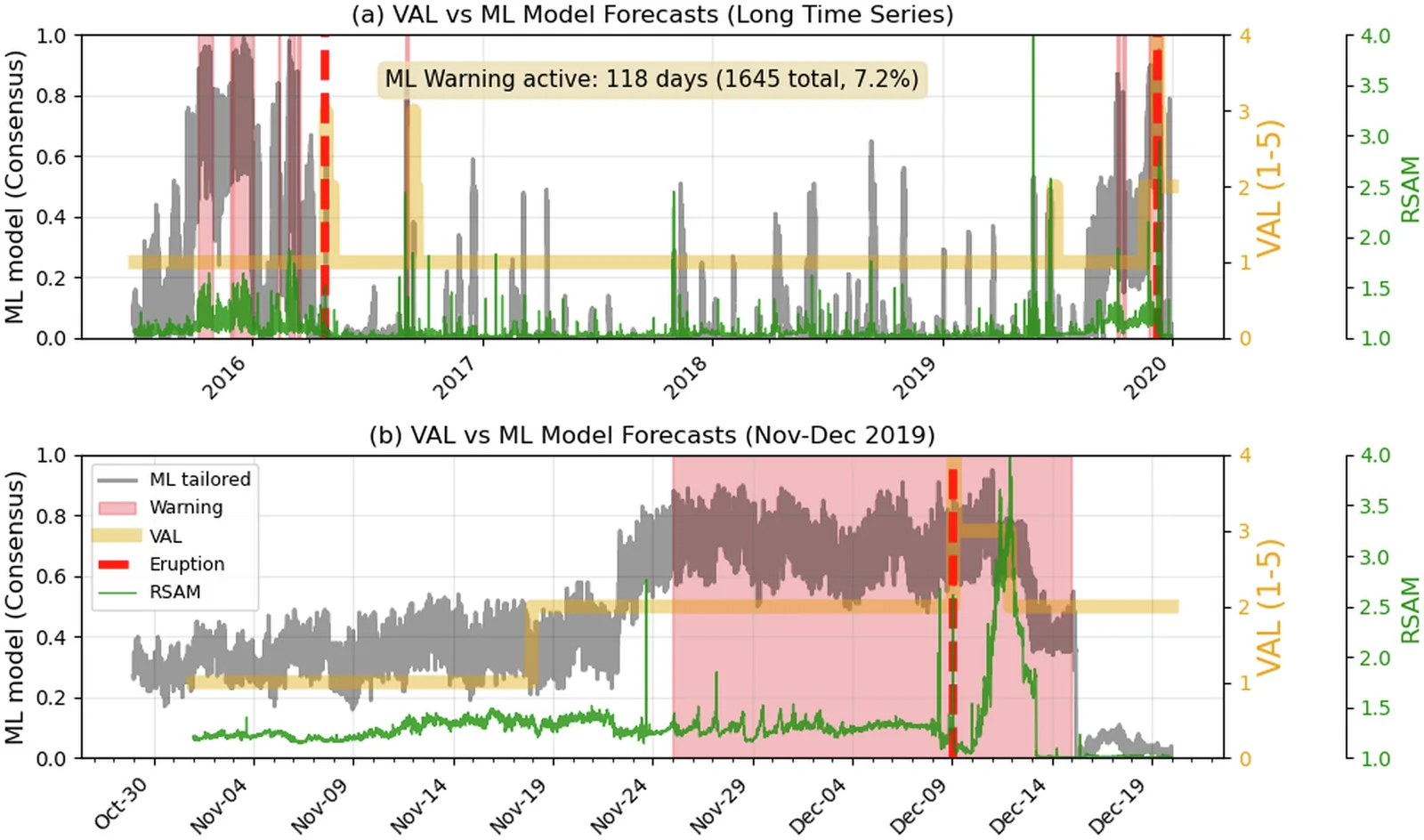
Comparison of ML eruption probability forecasts with operational alert levels at Whakaari (2015–2020). **a** Long-term context showing ML forecast probabilities (gray, left axis), RSAM seismic amplitude (green, right axis), and as reference official Volcanic Alert Level is plotted (VAL; orange, right axis). Red shaded regions indicate periods when the ML warning system would have been active using a threshold-and-hold scheme (τ = 0.7, 5-day hold; see text), totaling 118 days (7.2% of the monitoring period). Red dashed lines mark eruptions. **b** Detailed view of November-December 2019 showing ML forecast behavior before and during the fatal 9 December eruption. The threshold-and-hold post-processing converts high-frequency ML probability fluctuations into discrete warning periods (red shading), providing actionable alerts while limiting warning-active time.

Operationally, this behavioral framing must be supported by alert designs that translate high-frequency forecast outputs into warnings that are actionable but not overwhelming. Short-term forecasting tools (e.g., RSAM and machine-learning models) can fluctuate on much shorter timescales than established alert systems such as the Volcanic Alert Level (VAL^69^), reflecting sensitivity to transient seismic patterns (Fig. 4a). To bridge this mismatch, we apply a threshold-and-hold post-processing scheme^11^, in which alerts are triggered only when forecasts exceed a chosen threshold and remain active until elevated risk subsides for sustained periods (e.g., several consecutive days below threshold before deactivation).

In the Whakaari example, this approach reduces the proportion of time spent under active warning to 7.2% of days (118 of 1,645 days over 2015–2020; Fig. 4a), while preserving critical early-warning capability prior to the 2019 eruption (Fig. 4b). Such smoothing illustrates how false-alarm fatigue can be mitigated through alert design rather than by suppressing forecast sensitivity altogether. These parameters are illustrative; operational deployment requires calibration with agencies to balance competing objectives, including cost efficiency (maximizing PEV), behavioral response (maintaining compliance), and workflow compatibility (avoiding cognitive overload; in other words, operational deployment must remain simple enough to be useful for non-expert operators and should not add unnecessary layers of complexity). In this sense, PEV-informed forecasting functions as a decision-support and communication tool rather than a purely technical optimization.

Future extensions of the PEV framework could incorporate a dynamic trust variable that decays after false alarms and recovers after correct alerts, following the socio-hydrological warning model^70^. The ‘crying wolf’ hypothesis^71^ predicts that repeated false alarms erode public compliance, and some studies support this for tornado warnings^72^. However, others do not replicate the finding for protective behaviour^73,74^, and tsunami warning systems routinely operate with high false-alarm ratios while maintaining effective evacuation compliance, suggesting that warning credibility depends on risk communication practices and institutional trust rather than alarm frequency alone. Our threshold-and-hold post-processing scheme (Fig. 4) and the recommended reframing of alerts as risk indicators rather than binary predictions provide practical mechanisms for managing alert fatigue without requiring explicit trust modeling.

We acknowledge that this framework assumes that forecasts, once issued, lead to protective action. In practice, the translation of forecast to action depends on institutional decision-making, effective communication, and political acceptance of precautionary costs, particularly where tourism operators exert lobbying pressure (e.g., Whakaari, Copahue). The PEV framework supports this translation by providing quantitative evidence that forecast-based action yields net economic benefit, but successful implementation requires engagement with communities and civil authorities to build understanding of forecast uncertainty and acceptance that some alerts will not be followed by eruption.

### Model assumptions and data constraints

Our analysis focuses on direct, short-term economic impacts that can be reduced through early warning, including fatalities, injuries, emergency response, business interruption, and operational closures. We intentionally exclude longer-term consequences (e.g., infrastructure reconstruction, agricultural recovery, and national productivity decline), as these are largely unavoidable even under perfect forecasting. As a result, our estimates are conservative and likely underestimate the total benefits of effective forecasting, while ensuring that PEV captures only losses that are genuinely preventable through warning.

The cost model is necessarily simplified using binary outcomes (eruption/no eruption) and time-invariant parameters, whereas real losses vary with exposure, season, and behaviour. For example, exposure at Ruapehu peaks during the winter ski season ( ~ 10,000 people per day), while Whakaari hosted ~70 people per day on average during its operation period.

A natural extension of the framework is to replace the scalar missed-eruption loss L with a time-varying function L(t) that reflects seasonal or real-time exposure, following established practice in flood-damage assessment^75^ and hurricane warning decision models^76^. At Whakaari, for example, L(t) could be indexed to daily visitor manifests; at Ruapehu, to ski-field attendance. Because our fixed-L approach does not weight misses by exposure, the reported PEV values are conservative: dynamic weighting would increase estimated forecast value during peak-exposure periods when the consequences of a miss are greatest.

We do not address longer-term questions such as optimal monitoring network design, multi-hazard interactions, or integration with non-seismic data streams, nor do we explicitly model real-time exposure dynamics or graduated response strategies. Despite these limitations, consistent results across diverse volcanic settings demonstrate framework robustness, including stable optimal thresholds (τ ≈ 0.65–0.90) and positive PEV under order-of-magnitude cost uncertainty.

## Methods

The modeling methodology used here links eruption forecast outputs with short-term economic impact assessment using the Potential Economic Value (PEV) framework. By assigning costs to false alarms and missed eruptions and incorporating probabilistic severity distributions, we are able to compute PEV metrics for different forecast decision thresholds, evaluate how costs accumulate over time and are distributed across stakeholders.

### Potential economic value (PEV) Framework

Forecast utility is quantified by comparing expected costs under three strategies for taking precautionary measures, termed “acting.” Precautionary measures are a binary choice to evacuate (or not) and it is assumed that evacuation reduces loss-of-life exposure to zero. The three strategies evaluated are (1) acting imperfectly on the instruction of a forecast, (2) never acting, which is the same as having no forecast information on which to act, and (3) and acting perfectly, which assumes a forecast that is 100% accurate.

We consider forecasting systems that output a continuous numerical “risk of eruption” variable $y(t)\in [0,{y}_{\max }]$. A binary precautionary action is parameterized by a decision threshold τ, with an action (e.g., evacuation/closure) taken whenever $y(t)\ge \tau$.

Forecast performance at threshold τ is summarized using contingency statistics computed over fixed decision windows. We define, the miss fraction:

1 $$m\left(\tau \right)=1-{{{\rm{TPR}}}}\left(\tau \right),$$

where ${{{\rm{TPR}}}}\left(\tau \right)$ (True Positive Rate) is the fraction of pre-eruptive decision windows correctly flagged. The false-alarm burden over the evaluation horizon

2 $$b(\tau )={{{\rm{FPR}}}}(\tau )T,$$

where ${{{\rm{FPR}}}}(\tau )$ (False Positive Rate) is the fraction of non-event windows that exceed τ, and T is the number of non-event windows (or equivalently the duration of evaluated non-event time expressed in the same window units).

Under these definitions, the expected cost over the evaluation horizon when acting on the forecast at threshold τ is

3 $${E}_{{\mbox{forecast}}}\left(\tau \right)={Cb}\left(\tau \right)+{Lm}(\tau )$$

where C is the cost per false-alarm action (e.g., per day of closure) and L is the loss per missed eruption (expressed as a cost).

We compare this to two baselines: a no-forecast strategy that never acts, ${E}_{{\mbox{no}}}=L$ (i.e., all eruptions are missed in the binary-action sense), and a perfect forecast, ${E}_{{\mbox{perfect}}}=0$. Then the Potential Economic Value is then defined as

4 $${{{\rm{PEV}}}}(\tau )=\frac{{E}_{{{{\rm{no}}}}}-{E}_{{\mbox{forecast}}}\left(\tau \right)}{{E}_{{{{\rm{no}}}}}-{E}_{{\mbox{perfect}}}}=1-\frac{C\,b\left(\tau \right)+L\,m\left(\tau \right)}{L}.$$

PEV ranges from -∞ to 1, where:

- PEV = 1: perfect forecast (no false alarms, no missed eruptions)
- PEV = 0: forecast performs equivalently to no-forecast strategy
- PEV < 0: forecast causes more harm than doing nothing
- PEV > 0: forecast provides net benefit; magnitude indicates fraction of avoidable losses prevented

This formulation follows the standard cost–loss framework used to compute relative economic value in forecast verification^44,45^. Importantly, because PEV is a ratio of costs with consistent normalization, it is invariant to whether costs are expressed per decision window, per unit time, or over a fixed horizon, provided C, L, and the forecast performance terms are defined on the same basis.

As a framework, the PEV is dependent on the decision threshold τ, and therefore allows evaluation of different choices of τ. Furthermore, PEV is principally sensitive to a cost-ratio, C/L, rather than absolute magnitudes of individual cost contributions. As a dimensionless quantity, it is theoretically comparable across different locations and hazard types. Finally, it is directly evaluative of forecasts for decision-making contexts.

We applied PEV to a series of pseudo-prospective eruption forecasts that were obtained from a machine-learning model trained on seismic data from multiple volcanoes^19^. We tested the framework on five case study volcanoes excluded from the training set to compare the relative quality of these forecasts under different decision thresholds and cost assumptions.

The first two volcanoes were Whakaari, in New Zealand, and Mt Ontake, in Japan. For these examples, cost parameters are derived from documented casualties, economic impacts, and recovery expenses from recent fatal eruptions. Mt Ruapehu and Copahue, has not experienced a mass-casualty eruption. However, for PEV to be useful in justifying the introduction of an operating forecast system at a particular volcano, such hypothetical applications are necessary. In these situations, cost parameters are estimated based on historical precedents, regional economic data, and risk assessments, and then subject to sensitivity analysis.

This approach distinguishes evaluation cases, where PEV results can be compared to actual outcomes, from demonstration where PEV reveals forecast value under plausible but unconstrained future scenarios. PEV measures only the avoidable portion of eruption-related losses (those that can be mitigated through timely warning and protective actions) and therefore does not include physical damage that would occur even under perfect forecasting.

### Forecast data and model outputs

We evaluate a forecasting model that estimates a likelihood proxy of an eruption occurring within the next 48 h based on the preceding 96 h of seismic activity. These forecasts are derived from the model of Ardid & Dempsey et al. (2025)^19^, which extracts time series features from seismic data from a 24-volcano training dataset and outputs a continuous time series (“consensus”) sampled every 10 min between 0 and 1.

PEV analysis was applied to these forecast time series across extended eruptive and non-eruptive periods for each volcano. Forecasts were evaluated under out-of-sample conditions (pseudo-prospective forecasting^19^).

To translate continuous forecast outputs into operational decisions, a decision threshold (τ) was applied to the forecast likelihood time series. Values exceeding τ were interpreted as eruption alarms, while values below τ indicated no alarm. Alarms were evaluated relative to eruption catalogs using a fixed pre-eruptive window of 48 h prior to eruption onset. Alarms occurring outside these windows were classified as false alarms, whereas failures to raise an alarm before an eruption were classified as missed eruptions. By sweeping τ across the [0, 1] range, we quantified how false-alarm rates, missed-eruption rates, and cumulative costs vary with threshold choice, forming the basis of the PEV analysis (Details of model architecture, alarm construction, and pseudo-prospective evaluation are discussed in Ardid & Dempsey et al. (2025)^19^ and also provided in Supplementary Text S4).

### Cost assumptions

We applied the PEV framework to five volcanoes spanning diverse eruption frequencies, hazard styles, exposure patterns, and socio-economic contexts: Whakaari, Ruapehu, Ontake, Copahue, and Bezymianny. Costs represent short-term, forecast-sensitive impacts, including economic disruption from precautionary actions (false alarms; C) and representative human and economic losses from eruptions occurring without mitigation (missed eruptions; L).

Cost parameters were chosen using documented impacts from historical eruptions, official loss estimates, and established valuations of human life, supplemented by conservative assumptions where empirical data are incomplete. Across all cases, false-alarm costs are likely overestimated, while missed-eruption losses are likely underestimated (particularly for rare, high-consequence events with cascading effects) yielding a cautious lower bound on forecast value. This conservative bias ensures that positive PEV values do not depend on optimistic cost assumptions.

Uncertainty in cost estimates was explored using ±50–60% Monte Carlo variation, and volcano-specific justifications, references, and sensitivity analyses are provided in Supplementary Text S5 and S6.

For each volcano we specify central values of costs around which sensitivity is tested:

#### Whakaari (New Zealand)

- **False-alarm cost:** NZD 0.5 M per day (short-term disruption to commercial tourism operations, marine transport, and associated services during precautionary closures).
- **Missed-eruption cost:** USD 20 M (risk-weighted representation of human and economic losses from a Whakaari-style catastrophic event, distributed across multiple forecast cycles; see Supplementary Text S5.1).

#### Ontake (Japan)

- **False-alarm cost:** USD 1 M per day (temporary closure of summit trails, ropeways, and mountain facilities during peak hiking seasons).
- **Missed-eruption cost:** USD 400 M (human-capital losses from ~63 fatalities combined with prolonged post-eruption tourism and access restrictions).

#### Ruapehu (New Zealand)

- **False-alarm cost:** USD 0.5 M per day (ski-field closures and associated regional tourism disruption during winter operations).
- **Missed-eruption cost:** USD 75 M (conservative estimate informed by the 1953 Tangiwai lahar disaster and the 1995–96 eruption sequence).

#### Copahue (Chile/Argentina)

- **False-alarm cost:** USD 0.5 M per day (logistical and economic costs associated with mandatory evacuations and access restrictions, based on the 2013 crisis).
- **Missed-eruption cost:** USD 30 M (risk-weighted average combining plausible minor phreatic events and lower-probability catastrophic scenarios affecting nearby communities).

#### Bezymianny (Russia)

- **False-alarm cost:** USD 1 M per day (operational costs of precautionary airspace management and trans-Pacific flight rerouting).
- **Missed-eruption cost:** USD 250 M (aviation disruption and infrastructure losses associated with large ash-producing eruptions, informed by historical aircraft–ash encounters).

## Supplementary information


Supplementary Information
Transparent Peer Review file


## Source data


Source Data


## Data Availability

We provide the ML forecasting model output (consensus time series) for Whakaari over ~10 years, from which the PEV is computed. The data are provided in the inputs/ folder and include: (1) ML model consensus predictions in pickle format for both tailored (volcano-specific) and generalized (multi-volcano) models, containing eruption probability predictions at 10-minute intervals; (2) eruption times (5 eruptions from 2012-2019) in text format used for forecast evaluation; and (3) pre-processed seismic amplitude datasets (RSAM) sampled at 10-minute intervals with pre-computed ROC curve data. Raw seismic waveform data for New Zealand volcanoes are publicly available from GeoNet and can be accessed via standard FDSN web services. The datasets used in this study are accessible as FDSN clients through the International Federation of Digital Seismograph Networks (FDSN) web service infrastructure (https://www.fdsn.org/networks/). Source data are provided with this paper.

## Code availability

Code used to generate the ML forecasts are given in previous work (ref. ^19^). Here we provide the code to evaluate Potential Economic Value (PEV) from the ML forecasting model outputs. This includes scripts for threshold evaluation and cost-loss analysis. The main script (pev_model.py) contains functions for: (1) threshold evaluation (roc_counts()) that computes True Positive Rate (TPR) and False Positive Rate (FPR) across probability thresholds using pre-eruption windows; (2) cost-loss analysis (pev_curve()) that calculates PEV and net savings by comparing forecast costs with losses from missed eruptions; and (3) Monte Carlo uncertainty quantification (monte_carlo_pev()) for cost/loss parameter uncertainty. The code also includes visualization functions to generate ROC curves, PEV curves, and summary tables. Supporting files include example configuration templates, usage examples, documentation, and Python package dependencies. The code is designed to work with the provided Whakaari data and can be extended to other volcanoes by modifying the configuration.
