# Supplementary material for: Socio-economic value of data-driven eruption forecasts to balance false alarms against catastrophic loss
Source: Nat Commun. 2026 Aug 26;17:9356. doi: 10.1038/s41467-026-77242-4 (PMC13534426; doi:10.1038/s41467-026-77242-4)
Supplement: Supplementary file 1 [file 41467_2026_77242_MOESM1_ESM.pdf]

SUPPLEMENTARY INFORMATION

Socio-economic value of data-driven eruption forecasts to balance false alarms against catastrophic loss

Alberto Ardid[1*], David Dempsey[1], Garry McDonald[2], Shane Cronin[3]

[1]University of Canterbury, Christchurch, New Zealand, 2Market Economics, Auckland, New Zealand, 3University of Auckland, Auckland, New Zealand

*Corresponding author: Alberto Ardid, alberto.ardid@canterbury.ac.nz

Index

S1. Sensitivity analysis for Copahue
S1.1 Sensitivity to catastrophic-loss probability
S1.2 Sensitivity to alert decision threshold

S2. From scale to vulnerability: rethinking eruption impact

S3. Stakeholder allocation
Missed eruptions (high-impact losses)
False alarms (manageable disruptions)
Rationale and policy implications

S4. Forecast data and model outputs
Alarm issuance and forecast evaluation

S5. Cost assumptions
S5.1 Volcano-specific cost justifications
Whakaari / White Island (New Zealand)
Ruapehu (New Zealand)
Ontake (Japan)
Copahue (Chile/Argentina)
Bezymianny (Russia)

S6. Extended justification for cost assumptions

Supplementary Tables
Table S1. Eruption frequency and impact assumptions
Table S2. Tailored and generalized machine-learning forecast models

Supplementary Figures
Figure S1. Sensitivity of forecast value to catastrophic-loss probability (Copahue)
Figure S2. Sensitivity of forecast performance and value to alert threshold (Copahue)
Figure S3. Cumulative cost breakdown by stakeholder (Whakaari)

Supplementary References

Supplementary text

S1. Sensitivity analysis for Copahue

To assess the robustness of forecast value estimates for Copahue under uncertainty in both loss severity and operational decision-making, we conducted two complementary sensitivity analyses: (1) sensitivity to the assumed probability of catastrophic loss given a missed eruption, and (2) sensitivity to the choice of alert decision threshold. Copahue provides a useful test case because missed eruptions are not expected to dominate total losses under most plausible scenarios. Instead, expected impacts are more evenly balanced between precautionary-action costs and missed-eruption losses, making forecast value highly sensitive to both cost assumptions and threshold selection.

S1.1 Sensitivity to catastrophic-loss probability

In the first analysis, we explored how forecast value responds to uncertainty in the probability that a missed eruption results in catastrophic loss, $P(\text{catastrophic loss} \mid \text{miss})$. While the baseline Copahue configuration assumes a risk-weighted value of 0.20 (Section S1.1), we varied this probability across the full range from 0 to 1. For each value, we generated a stochastic no-forecast baseline ensemble (n = 100 realizations), in which each missed eruption draws either a minor or catastrophic loss according to the specified probability.

Figure S1 illustrates how relative and absolute savings, baseline costs, and expected loss per missed eruption vary with $P(\text{catastrophic loss} \mid \text{miss})$. As expected, baseline costs increase approximately linearly with increasing catastrophic-loss probability, reflecting the growing contribution of rare high-impact outcomes. In contrast, forecast costs remain fixed, as the forecast path is unchanged. Consequently, relative and absolute forecast savings increase rapidly once catastrophic outcomes become plausible.

A key result is that forecast value becomes positive at relatively low catastrophic-loss probabilities and remains strongly positive across a wide parameter range. This behaviour reflects the asymmetric cost structure of eruption forecasting: even when catastrophic outcomes are infrequent, avoiding a small number of high-consequence misses dominates the cumulative cost of frequent but lower-impact false alarms.

S1.2 Sensitivity to alert decision threshold

In the second analysis, we examined sensitivity to the alert probability threshold (τ), which governs the trade-off between false alarms and missed eruptions. Using a fixed catastrophic-loss probability of $P(\text{catastrophic loss} \mid \text{miss}) = 0.20$, we evaluated forecast performance and economic outcomes across a range of thresholds from τ = 0.30 to 0.90.

Figure S2 shows how false alarms decrease monotonically with increasing threshold, while missed eruptions increase, illustrating the fundamental trade-off inherent in threshold-based warning systems. Intermediate thresholds reduce false alarms substantially while still capturing a majority of eruptions, whereas high thresholds suppress alerts almost entirely at the expense of multiple missed events.

Economic performance follows this trade-off. Relative savings peak at intermediate thresholds (τ ≈ 0.6–0.65), where the cumulative cost of false alarms is outweighed by the reduction in missed-eruption losses. Positive forecast value is not confined to a single optimal threshold but persists across a broad range of intermediate values. At very high thresholds, forecast costs exceed the no-forecast baseline, as even a small number of missed eruptions erodes economic benefit.

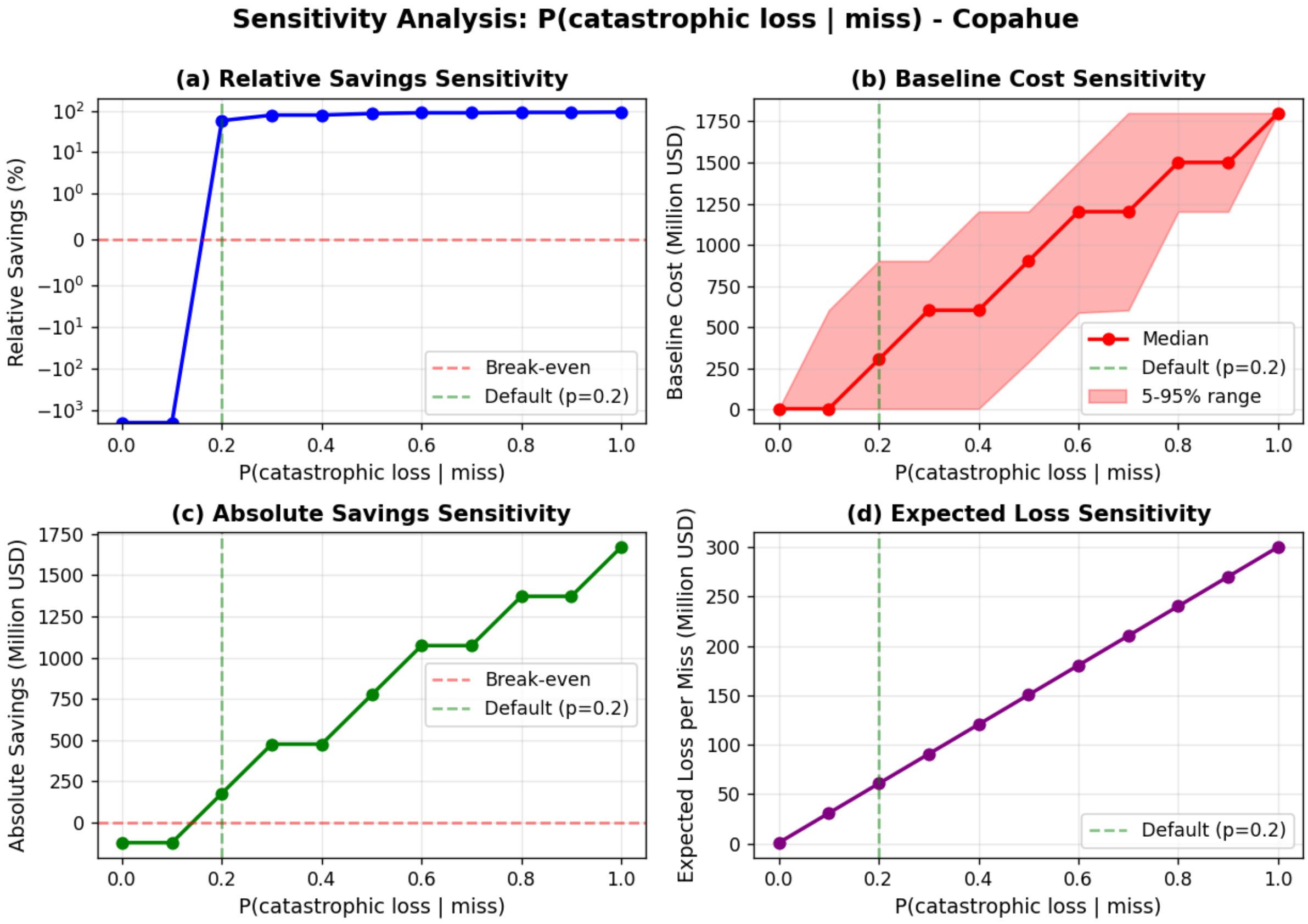

Figure S1. Sensitivity of forecast value at Copahue to the assumed probability of catastrophic loss given a missed eruption. Sensitivity of relative savings (a), baseline cost (b), absolute savings (c), and expected loss per missed eruption (d) to P(catastrophic loss | miss). Baseline costs are estimated using a stochastic no-forecast ensemble (n = 100 realizations), with shaded regions in (b) indicating the 5–95% range. The forecast cost is held fixed, as the forecast path does not depend on loss severity. Vertical dashed lines mark the baseline assumption $P(\text{catastrophic loss} \mid \text{miss}) = 0.20$. Results show that forecast value becomes positive at relatively low catastrophic-loss probabilities and increases rapidly as the likelihood of high-impact outcomes grows, reflecting the dominance of rare but severe missed eruptions over frequent low-cost false alarms.

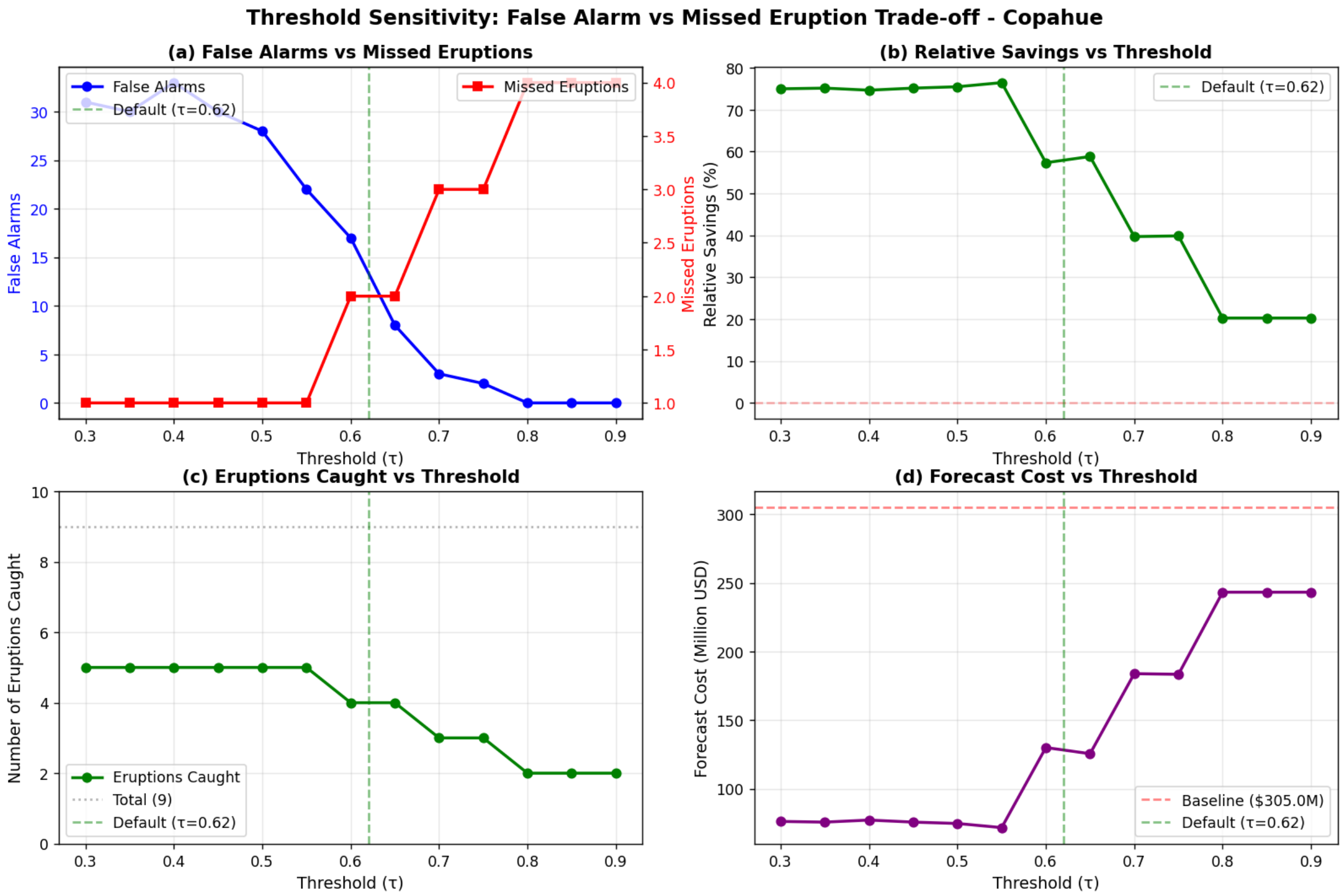

Figure S2. Sensitivity of forecast performance and economic value at Copahue to the alert decision threshold. Sensitivity of false alarms and missed eruptions (a), relative savings (b), number of eruptions caught (c), and total forecast-related cost (d) to the probability threshold τ, evaluated at $P(\text{catastrophic loss} \mid \text{miss}) = 0.20$. The dashed vertical line marks the threshold that maximizes expected economic value under the assumed cost structure. Forecast value is positive across a broad range of intermediate thresholds, where reductions in missed-eruption losses outweigh the cumulative cost of false alarms, while very high thresholds lead to negative value due to increased missed eruptions.

S2. From scale to vulnerability: rethinking eruption impact

Typically, the Volcanic Explosivity Index (VEI[1]) is used to classify eruptions by size and energy release, yet the true impact of an event depends more on exposure and vulnerability than on physical magnitude. Ontake in 2014 (63 fatalities[2]) and Whakaari in 2019 (22 fatalities and dozens of life-altering injuries[3]) were both low on the VEI scale (VEI 2), illustrating that small eruptions can still result in severe human consequences. These events highlight the life-safety risk at "touristic volcanoes," where many unprepared visitors are concentrated near craters or summits[4]. In such settings, vulnerability is amplified by the absence of timely warning, limited evacuation options, and the difficulty of interpreting weak or short-lived precursors[5,6]. Recognizing that human and economic losses scale primarily with exposure—not eruption size—underscores the need for integrated physical and socioeconomic approaches to risk management[7,8].

This pattern extends beyond individual touristic sites to entire urban regions built within volcanic zones, where population growth and economic activity increasingly overlap with areas of persistent unrest. Many of these recent disasters occurred at touristic volcanoes, where high

visitor exposure amplifies vulnerability even during small or moderate eruptions[9,10]. The 2010 Merapi eruption demonstrated the effectiveness of timely evacuations for more than 400,000 people, yet also illustrates how precautionary or false-alarm evacuations can entail significant short-term economic disruption; an issue especially relevant for cities built on or near volcanoes that combine high population, ongoing unrest, and clear economic or logistic vulnerability, with recent work identifying 381 cities and >118 million people living within 30 km of an active volcano. These include, for example, Manila in the Philippines (Taal/Pinatubo), Jakarta in Indonesia (Merapi and surrounding fields), Mexico City (Popocatépetl), Quito in Ecuador (Pichincha), Goma in the Democratic Republic of the Congo (Nyiragongo), Naples in Italy (Campi Flegrei/Vesuvius), Auckland in New Zealand (Auckland Volcanic Field) and/or Kagoshima in Japan (Sakurajima). Importantly, while the economic consequences of natural hazards and evacuations are typically concentrated at local scales and often short-lived, tourism and economic activity tend to shift rather than disappear at national levels (e.g., Kaikōura earthquake, New Zealand[11]).

S3. Stakeholder allocation

We distributed false alarm costs and missed-eruption losses across stakeholder groups to reflect the relative burdens borne during volcanic crises. This analysis focuses on Whakaari (New Zealand), using the 2019 eruption as a reference point to inform cost magnitudes and allocations. While approximate, the allocation is grounded in reported figures from that event and serves to illustrate how losses may be distributed across stakeholder groups. Overall, false alarms predominantly impact private operators and the public through lost revenue and disruptions, whereas missed eruptions impose catastrophic consequences primarily on victims and the state.

Missed Eruptions (High Impact): We assume a central estimate of USD 100 million in total loss for Whakaari, intended to represent the combined scale of reparations, health impacts, and reputational damage. This value is consistent with the broader Cost Assumptions framework (see previous subsection), where we estimated Whakaari's 2019 eruption at ~USD 100 million in direct and indirect losses, including 22 fatalities, 25 severe injuries, and substantial economic disruption[12].

For illustrative allocation, we distributed this total as:

- **60% to victims and families,** reflecting the dominant weight of fatalities, severe injuries, and long-term health burdens. Using New Zealand's social cost of NZD 4.42 M per fatality (USD ~2.7 M) and NZD 467,700 per serious injury (USD ~290,000)[13], the 22 fatalities and 25 injuries already imply ~USD 85 M in human costs, consistent with this majority share.
- **25% to government agencies,** covering emergency response, medical treatment, investigations, and legal settlements. WorkSafe's investigation alone exceeded NZD 5.5 M in direct costs[14].
- **15% to the tourism industry,** capturing long-term reputational losses, cancellations, and operator closures. Following the eruption, New Zealand courts ordered more than NZD 10 M in compensation (USD ~6.1 M) and ~NZD 2.6 M in fines for tour operators[15].

These proportions are not directly measured but are grounded in the scale of documented costs and align with the simplified loss parameters defined in the Cost Assumptions section. Valuations from international benchmarks (e.g., OECD[16], U.S. Department of Transportation[17]) suggest that our estimates remain conservative; valuing Whakaari's 22 fatalities at U.S. DOT levels (~USD 12.5 M each) would imply >USD 250 M in human losses alone.

False Alarms (Manageable Disruptions): For Whakaari, we assume a notional cost of USD 0.5 million per false alarm event, reflecting realistic orders of magnitude for operational disruptions such as tour cancellations, ticket refunds, and lost revenue. This value is consistent with the Cost Assumptions framework (see previous subsection), where daily losses to the tourism sector and associated services were estimated at NZD ~0.5 million during precautionary closures.
We allocate this total across stakeholders as follows:

- **50% to tourism operators**, reflecting the dominant share of direct disruptions. Operators faced more than NZ$10 million in reparations and fines after the 2019 eruption, highlighting their financial exposure during volcanic crises. Even in non-eruption scenarios, lost ticket sales and refunds create recurring burdens concentrated on operators.
- **30% to government agencies**, representing the costs of mobilizing emergency services, enforcing closures, and conducting regulatory oversight. WorkSafe's investigation into the eruption exceeded NZ$5.5 million in legal and operational expenses, illustrating the scale of government involvement in volcanic crisis management[14].
- **20% to the public**, capturing smaller, diffuse costs such as disrupted itineraries, transport changes, and alternative accommodation. While less individually severe and less directly documented, broader studies show that elevated volcanic alerts can generate **short-term indirect effects** on travel and local economic activity without long-term damage (e.g.[18,19]). This share acknowledges those impacts without overstating their scale.

This 50 : 30 : 20 split is therefore presented as a **representative allocation**, grounded in the relative magnitudes of documented costs while recognizing that empirical data on false alarms remain limited.

Rationale and Policy Implication: This decomposition illustrates that forecasting can reduce overall societal losses by shifting burdens: false alarms impose recurring but manageable costs, largely on private operators and individuals, while missed eruptions lead to rare but catastrophic losses borne mainly by victims and the public sector. This framing underscores that governments cannot deflect severe risk to private entities, yet private actors may reasonably absorb smaller, predictable disruptions.

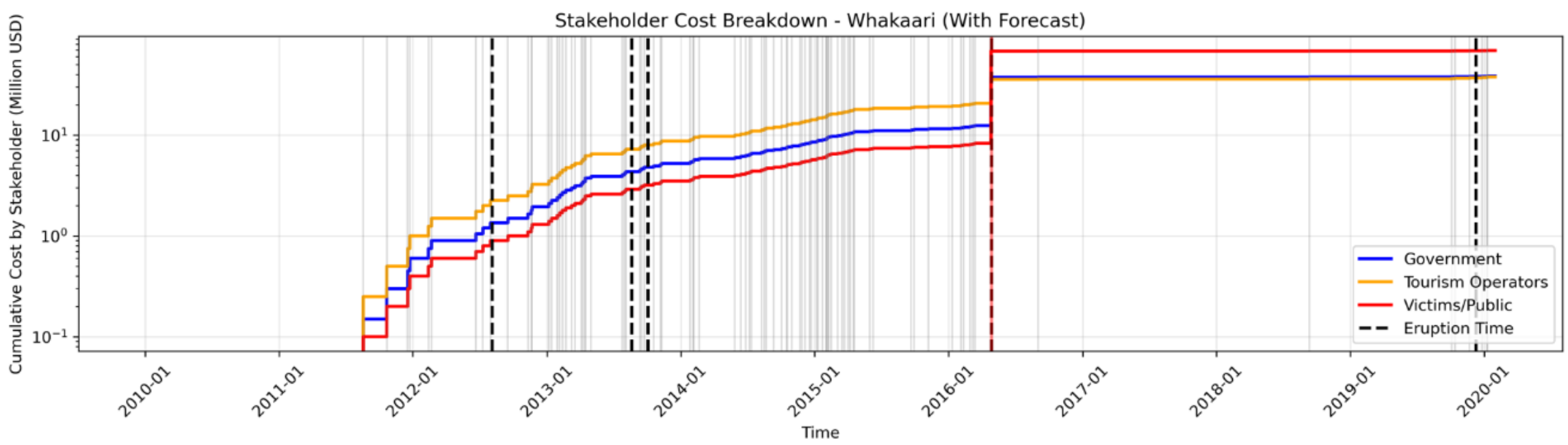

Figure S3. Cumulative Cost Breakdown for Whakaari Volcano by Stakeholder (With Forecast). Cumulative economic costs for Government (blue), Tourism Operators (orange), and Victims/Public (red) related to Whakaari volcano from 2010-2020. Historical data shows Tourism Operators bearing the highest costs (~$15-20M), followed by Government (~$10-12M) and Victims/Public (~$8-10M). Black dashed lines mark eruption events with associated cost jumps. The red dashed line around 2016 indicates transition to a forecast period where costs stabilize. Gray lines represent minor events contributing to gradual increases. The forecast system demonstrates potential to mitigate future cost escalations across all stakeholder groups.

S4. Forecast data and model outputs

We evaluated machine-learning forecast outputs that estimate eruption likelihood in the upcoming 48 hours based on seismic data received in the previous 96 hours. Specifically, we use the model results of Ardid & Dempsey et al.[12], which combine seismic features extracted from a training dataset of 24 volcanoes. Forecasts were assessed under out-of-sample conditions, meaning that eruptions used for testing were excluded from model training, thereby simulating pseudo-prospective forecasting[12].

The forecasts derived from seismic records analysed over different durations and eruption histories across the case study volcanoes (see Ardid & Dempsey et al.[12]). At Whakaari (New Zealand), we used data from station WIZ (500 m from the crater) covering 11 years between 2008 and 2019, encompassing five phreatic eruptions (2012, two in 2013, 2016, and 2019). Ruapehu (New Zealand) was represented by station FWVZ (2.5 km), with a 14-year record that includes three phreatic eruptions in 2006 and 2007. For Ontake (Japan), station ONTA (2 km from the summit) provided ~1.5 years of data leading up to the 2014 phreatic eruption. Copahue (Chile/Argentina) was monitored from station COP (4.5 km), with 1.5 years of record during which three phreatic eruptions occurred in 2020. Bezymianny (Russia) was represented by station BELO (1 km), with a 3-year dataset spanning three magmatic eruptions in 2007–2009.

From these seismic records, consensus forecast time series were generated from tailored and generalized ML models (see Table S2[12]). These eruption likelihood traces are continuous in time at 10-minute resolution, allowing us to evaluate false alarms, missed eruptions, and cumulative costs relative to eruption catalogs.

Alarm issuance and forecast evaluation

Forecast model outputs are continuous likelihood values between 0 and 1. To translate

these traces into binary alarms, a decision threshold is applied: values exceeding the threshold are interpreted as an "eruption alarm," while values below indicate no alarm. Alarms are then evaluated against eruption catalogs using standard classification metrics (Fig. 6[12]). True positives (TP) occur when an alarm is raised within the pre-defined pre-eruptive window (48 h before an eruption onset), and true negatives (TN) when no alarm is raised during non-eruptive periods. False positives (FP) are alarms outside pre-eruptive windows, representing spurious alerts, while false negatives (FN) are failures to raise an alarm before an eruption. By sweeping thresholds across the [0–1] range, we quantify forecast skill using Receiver Operating Characteristic (ROC) curves, from which true positive rates (TPR = TP/(TP + FN)) and false positive rates (FPR = FP/(FP + TN)) are calculated. This procedure ensures alarms are assessed systematically across multiple thresholds, enabling evaluation of false-alarm rates, missed events, and cumulative costs in a pseudo-prospective manner.

S5. Cost Assumptions

We applied the PEV framework to six volcanoes (Whakaari, Ruapehu, Ontake, Copahue, and Bezymianny), chosen to span diverse eruption frequencies, magnitudes, hazard styles, and socio-economic contexts[12]. Eruption onset times were compiled from observatory bulletins and eruption catalogs (e.g., Global Volcanism Program).

Our cost framework isolates **short-run, flow-dominated impacts** (closures, transport restrictions, visitor losses, response mobilization) supplemented by **representative stock losses** (fatalities, injuries, asset destruction) where relevant. We exclude longer-term general-equilibrium effects such as structural economic shifts, price changes, or multi-year labor impacts[20,21]. Thus, values presented here are **conservative, stylized estimates**, enabling cross-volcano comparison rather than full economic appraisal.
Cost parameters are expressed as **false-alarm cost (C)** per day of precautionary action and **missed-eruption cost (L)** for an eruption occurring without mitigation. Each central estimate is varied by ±50–60% in Monte Carlo simulations, consistent with uncertainty in valuing human life[13,16,17], indirect economic impacts, and differing societal risk tolerances[22,23,24].

S5.1 Volcano-Specific Cost Justifications

Whakaari / White Island (New Zealand)

False alarm (C): NZD 0.5 million per day.
Whakaari's economy is dominated by tourism, with commercial tours, licensing agreements, and associated services (food, accommodation, marine transport). Ngāti Awa experienced a **NZD 1.4 M annual loss** following the 2019 eruption closure. Licensing fees alone total **hundreds of thousands NZD per year**, and daily revenue for operators often reaches **tens of**

thousands NZD. When regional spillovers are included, NZD 0.5 M/day is a plausible, conservative figure.

Missed eruption (L): USD 20 million.

Economic losses from the 2019 eruption are estimated at **~USD 100 M**, including fatalities, injuries, restitution, fines, and immediate response.

- 22 fatalities, 25 severe injuries.
- Restitution and fines ≈ NZD 12.6 M (~USD 6.1 M).
- Response costs ≈ NZD 4.9 M (~USD 3 M).
- Combined ≈ USD 190k per victim (conservative).
- Not included: long-term rehabilitation, litigation, SAR operations, reputational harm, tourism decline.

New Zealand's **Value of Statistical Life (VSL)** is NZD 4.42 M (USD ~2.7 M) per fatality and USD 290k per serious injury[13].

International values range **USD 3–12.5 M per fatality**[16,17].

Applying U.S. values to Whakaari's 22 fatalities implies **USD >250 M** in human losses alone.

To avoid overweighting a single catastrophe, we distribute the USD 100 M loss over **five cycles** (four years with minimal impacts + one catastrophic event), yielding **L ≈ USD 20 M**.

Citations: [13,16,17,22].

Ruapehu (New Zealand)

False alarm (C): USD 0.5 million per day.

Ruapehu's economy relies on winter tourism:

- Visitor expenditure ≈ **NZD 100 M/year**[25].
- Ohakune GDP ≈ **NZD 74 M**, dominated by recreation, accommodation, and retail linked to ski operations[26].

Even short closures ripple through local businesses, making USD 0.5 M/day a conservative order-of-magnitude estimates.

Missed eruption (L): USD 75 million.

Two historical references:

1. **1953 Tangiwai lahar**: 151 fatalities[27,28].
2. **1995–96 eruptions**: losses > NZD 130 M across ski tourism, hydropower, agriculture, and aviation[29].

Using NZ VSL (NZD 4.42 M ≈ USD 2.7 M), the Tangiwai fatalities alone imply **USD 410 M** excluding injuries. OECD[16] and USDOT[17] values would be even higher. Thus, USD 75 M is intentionally conservative.

Citations: [29,27,25,13,16,17].

Ontake (Japan)

False alarm (C): USD 1 million per day.
Ontake's summit trails, ropeways, lodges, and cafés attract large seasonal visitor flows. Comparable volcanic tourism disruptions (e.g., Vulcano Island) have resulted in **80% annual turnover losses** during high-season evacuations[10]. Thus USD 1 M/day is an upper-limit but plausible value.
Missed eruption (L): USD 400 million.
Based on:

- **63 fatalities / missing**[2].
- Japan's VSL ≈ USD 4–8 M[30] → human losses alone **USD 250–500 M**.
- Near-decade closure of summit facilities causing prolonged economic paralysis.

Thus, USD 400 M is a conservative upper bound.

Citations: [2,30,10].

Copahue (Chile/Argentina)
False alarm (C): USD 0.5 million per day.
Based on 2013 mandatory evacuation of ~2240 residents[31], involving military transport, shelter, and multi-day operations.
Missed eruption (L): USD 30 million.
Risk-weighted average:

- 10% catastrophic (USD 300 M).
- 90% minor phreatic (USD 1 M).
  $L = 0.1 \times 300M + 0.9 \times 1M \approx$ USD 30 M.

Citations: [31].

Bezymianny (Russia)

False alarm (C): USD 1 million per day.
Frequent eruptions along trans-Pacific flight corridors → diversions and ash avoidance operations cost tens of millions per incident[32].
Missed eruption (L): USD 250 million.
Justification:

- 1956 lateral blast comparable to St. Helens[33].
- 129 documented aircraft–ash encounters (1953–2009) caused >USD 250 M in damage[34].

Thus, USD 250 M is modest but defensible.

Citations: [33,32,34].

* Expanded cost rationales are provided in Section S5.

S6. Extended Justification for Cost Assumptions (Supporting S1.1)

This section provides detailed support for the cost assumptions summarised in Section S1.1.

For each volcano, we parameterized the cost of a false alarm (C) and a missed eruption (L) based on historical impacts (direct economic loss) and published assessments:

Whakaari (New Zealand): We assumed false alarms would cost approximately NZD 0.5 million per day, reflecting disruption to tour operations and associated business closures. Licensing alone for tour access cost hundreds of thousands of NZD annually, while tourism-related revenue losses (e.g., Ngāti Awa's NZD 1.4 million annual drop in income) support a daily cost in the tens of thousands of NZD. When broader regional impacts (indirect loss) across operators and services are included, a figure of NZD 0.5 million per day is plausible. We estimate total economic losses from the 2019 eruption at approximately USD 100 million, which supports our simplified "missed eruption cost" of USD ~20 million per major event. A total of 47 individuals were present, resulting in 22 fatalities and 25 severe injuries. Combined restitution and fines (~NZD 12.6 million or USD ~6.1 million) plus response costs (~NZD 4.9 million or USD ~3 million) equate to an average of ~USD 190 k per person. This provides a conservative baseline, as many other costs were not included (such as long-term medical treatment and rehabilitation, litigation, search-and-rescue operations, reputational damage to New Zealand tourism, and regional economic disruption). No general-equilibrium effects have been accounted for; that is, the wider economy is expected to recalibrate over time through price and substitution adjustments following the event. The missed-eruption cost of USD ~20 million is derived by spreading one catastrophic event (estimated at ~USD 100 million in total losses) evenly across five forecast cycles (four minimal-impact, one catastrophic). For perspective, New Zealand's social cost per fatality (loss modelling) is estimated at NZD 4.42 million (USD ~2.7 million) and per serious injury at NZD 467,700 (USD ~290,000)[13]. International benchmarks are higher: OECD[16] guidance suggests USD 3–9 million per fatality, and the U.S. Department of Transportation[17] currently applies ~USD 12.5 million. Valuing Whakaari's 22 fatalities at U.S. levels would imply >USD 250 million in human losses alone, underscoring the conservatism of our USD 20 million baseline.

Ruapehu (New Zealand): We estimated the cost of a false alarm at approximately USD 0.5 million per day to reflect the short-term but significant disruptions from precautionary ski field closures and transport interruptions. This figure is deliberately conservative and illustrative yet grounded in the economic context of the region (e.g., if total seasonal revenues were considered over the shorter effective ski period, rather than an annualized approximation, the daily cost could be substantially higher). Visitor spend from the ski fields in Ruapehu District contributes substantially to the local economy-approximately NZ$100 million in visitor expenditure per year, supporting

hundreds of jobs and regional services[25]. Ohakune, the nearby town that serves as the primary gateway to Ruapehu's ski terrain, recorded a GDP of about NZ$74 million in the year ending March 2021, with a considerable share derived from arts, recreation, accommodation, and retail services tied to winter tourism[26]. Because GDP measures only value added, approximately 40–45% of total gross output, it provides a conservative indication of the scale of economic activity linked to winter tourism, and actual gross output losses during closures would likely be higher. These figures illustrate the deep economic reliance on ski operations, meaning that even brief closures could ripple through local businesses. Thus, assigning a USD 0.5 million cost per false alarm acknowledges disruption effects while remaining a conservative order-of-magnitude estimates in the analytical framework. We set a missed-eruption cost of USD 75 million, informed by two historical precedents. First, the 1953 Tangiwai lahar disaster incurred 151 fatalities[27,28]. Second, the 1995–96 eruption sequence generated economic losses estimated at NZ$130 million or more[29], affecting ski tourism, hydropower, agriculture, and aviation sectors. In terms of human cost valuation, applying New Zealand's social cost per fatality of NZ $ 4.42 million (~USD 2.7 million) results in a theoretical human-cost burden of approximately USD 410 million for the 22 fatalities, excluding injuries and indirect economic impacts. International benchmarks, such as the OECD[16]'s USD 3–9 million value-of-statistical-life range, would imply substantially higher totals; however, these are treated as part of the sensitivity analysis rather than the baseline scenario. This shows that our USD 75 million estimate is intentionally conservative and serves primarily to illustrate the PEV framework rather than provide a full-scale economic estimation.

Ontake (Japan): A conservative upper-limit estimate of approximately USD 1 million per day is used to represent the economic disruption caused by short-term closures of summit trails, ropeways, mountain lodges, and cafés during peak hiking season. While no specific studies quantify Ontake's per-day tourism losses, analogous volcanic tourism disruptions provide context. For example, on Vulcano Island (Italy), a high-season evacuation of 3 months can lead to a loss of about 80% of the annual tourism turnover[10]. The missed-eruption cost is estimated at USD 400 million, encompassing both the tragic human losses and the prolonged socio-economic fallout. A key component is the valuation of human casualties: Japan's Value of a Statistical Life (VSL) is estimated at approximately ¥600 million to ¥1.2 billion (USD 4 million–8 million per life) on average[30]. With around 63 climbers killed or missing[2], the human-capital cost alone ranges from roughly USD 250 million to over USD 500 million. This alone justifies the upper-limit characterization of the USD 400 million estimate. Added to that is the prolonged economic disruption: summit facilities (including trails, ropeways, lodges, and cafés) remained effectively closed for almost a decade post-eruption, paralyzing the local tourism economy. Anchored in both the human-capital losses and extended

economic shutdown, the USD 400 million estimate stands as a conservative, credible upper bound for the trajectory of a missed eruption.

Copahue (Chile/Argentina): We estimated the false-alarm cost at approximately USD 0.5 million per day, based on the precedent set by the 2013 mandatory evacuation of around 2,240 residents within a 25-km radius around Copahue[31]. According to ONEMI[31]'s alert bulletin, army trucks and buses were deployed to evacuate these residents over several days, illustrating the significant logistical expenditures tied to evacuation operations (transport, shelter, staffing, and local disruptions). For a missed eruption, we assigned a risk-weighted expected loss of approximately USD 30 million, combining a 10% probability of a catastrophic scenario (estimated at USD 300 million in damages, e.g., devastation to Caviahue ski resort and adjacent towns) with a 90% probability of a minor phreatic event (costed at USD 1 million in local cleanup and disruption). This methodology yields a balanced, plausible average that accounts for both extremes of potential impact.

Bezymianny (Russia): We estimated the daily cost of a false alarm at USD 1 million, reflecting the potential economic impact of precautionary trans-Pacific flight diversions to avoid volcanic ash hazards. Although specific cost figures for those diversions are rare, the ash can severely damage aircraft systems, often requiring costly rerouted flights or maintenance, where estimates suggest such encounters can cost tens of millions of dollars each[32]. Given Bezymianny's frequent eruptions and its location along major air routes, this range of USD 01 million per day for diversion-related disruptions is a conservative yet plausible approximation for modeling purposes. For missed eruptions, we set a cost for missed eruptions at USD 250 million. Historically, the 1956 eruption of Bezymianny was a colossal lateral-blast event comparable in magnitude to Mount St. Helens' 1980 eruption[33], but occurred in a remote area, resulting in no direct human fatalities. This supports the focus on infrastructural and aviation-related consequences, which defines the financial scope of our "missed-eruption" cost mode. We also considered cumulative data: 129 reported aircraft–ash cloud encounters between 1953 and 2009, with numerous incidents involving engine, avionics, or structural damage has caused estimated monetary damage inflicted more than USD 250 million in aircraft damage[34]. This establishes a reasonable baseline for the overall risk associated with a major airborne ash event, making the USD 250 million figure a modest but defensible estimate.

Supplementary Tables

Table S1. Eruption frequency and impact assumptions used in the economic value analysis. Frequencies are based on published eruption histories where available; impact estimates combine reported economic losses and human costs, supplemented

by internal estimates when public data were unavailable. These values provide baseline parameters for stochastic ensembles and cost–loss evaluations across case study volcanoes.

Volcano	Eruption Frequency (episodes per decade)	Impact (economic/human costs)	Notes
Whakaari (NZ)	Frequent: 5 in out decade record in 2012, 2013, 2016, 2019. Historically, ~2 per decade.	~22 fatalities + tourism disruption; economic impact estimates not in public literature	Eruption activity 1975–2000, plus intermittent in 2012,2013, 2016, 2019. 2019 eruption fatalities.
Ruapehu (NZ)	Frequent: 2 in our 2 decade record in 2006, 2007. Historically, lahar events and eruptions every 1–3 yrs per crater lake eruption history.	Socio-economic impacts of 1995–96 eruption substantial; no exact USD published.	The 1953 Tangiwai disaster is the most famous lahar-related tragedy at Ruapehu, resulted in 151 fatalities and major rail disruption.
Ontake (Japan)	Rare; phreatic eruptions noted in 1979, 1991, and the deadly 2014 eruption	~63 climbers killed or missing in 2014.	2014 eruption: sudden phreatic explosion with no clear precursors. Summit facilities (trails, ropeways, lodges, cafés) remained largely closed for nearly a decade.
Copahue (Chile/Argentina)	Intermittent; eruptions recorded in 2012–2013, 2016, 2020 (6).	Missed-eruption cost risk-weighted at ~USD 30 M, balancing catastrophic scenarios (USD 300 M) vs minor phreatic events (~USD 1 M).	False-alarm cost estimated at ~USD 0.5 M/day (based on 2013 mandatory evacuation of ~2,240 residents within 25 km radius[31]).
Bezymianny (Russia)	Frequent; multiple eruptions each decade.	Missed-eruption cost ~USD 250 M, reflecting aviation losses and historic large eruptions.	1956 eruption comparable to Mount St. Helens33. 129 aircraft–ash encounters (1953–2009) caused >USD 250 M in damage34.

Pavlof, Redoubt, Augustine, Okmok, Veniaminof (Alaska, USA),	Mixed (some frequent eruptions, some rare);	Internal estimates from aviation disruption and eruption records	Redoubt cost ~USD 160 M (1989–1990), aviation > USD 101 M.
Eyjafjallajökull (Iceland)	Rare; notable eruption in 2010	Airline industry lost approx. USD 1.7 billion; total global economic impact estimated at up to USD 5 billion.	European airspace closed ~6 days; ~100,000 flights canceled affecting ~10 million passengers
Puyehue–Cordón Caulle	Intermittent; major eruptions in 1960 and 2011–2012	Airlines losses: ~A$30 M, tourism losses ~A$15 M; cleanup & relief funding ~US$2.41 B.	Ash cloud disrupted Southern Hemisphere flights; ~3,500 evacuated; widespread environmental impacts (evacuation, agriculture, infrastructure).
Tongariro (NZ)	Infrequent; small eruptions in 2012 (Te Maari vents), previous 1897, 1926–27	Economic costs mainly from aviation disruption and Tongariro Alpine Crossing closures; no fatalities in 2012	2012 eruption forced cancellation of flights and temporary closure of the Alpine Crossing, impacting tourism operators. Long historical dormancy punctuated by minor explosive episodes.

Table S2. Tailored and generalized machine learning (ML) models used in the eruption forecasting study (for more details see Ardid & Dempsey et al.[12]). Tailored models are trained on data from a single volcano, while generalized (cross-volcano) models are trained on multi-volcano datasets (out-of-sample, no data from the target volcano used in the training).

Volcano	Code	Model Type	Model Name	Training Data	Description
Whakaari	WIZ	Tailored	cve_WIZ	Whakaari only	Single-volcano model trained exclusively on Whakaari data

Whakaari	WIZ	Generalized	cvv_WIZ_FWVZ_KRVZ_ONTA_COP_SHW_BELO	Multi-volcano (Whakaari, Ruapehu, Tongariro, Ontake, Copahue, St Helens, Bezymianny)	Cross-volcano model trained on 6 volcanoes
Bezymianny	BELO	Tailored	cve_BELO	Bezymianny only	Single-volcano model trained exclusively on Bezymianny data
Bezymianny	BELO	Generalized	cvv_WIZ_FWVZ_KRVZ_ONTA_COP_SHW_BELO	Multi-volcano (Whakaari, Ruapehu, Tongariro, Ontake, Copahue, St Helens, Bezymianny)	Cross-volcano model trained on 6 volcanoes
Copahue	COP	Tailored	cve_COP	Copahue only	Single-volcano model trained exclusively on Copahue data
Copahue	COP	Generalized	cvv_WIZ_FWVZ_KRVZ_ONTA_COP	Multi-volcano (Whakaari, Ruapehu, Tongariro, Ontake, Copahue)	Cross-volcano model trained on 4 volcanoes
Ruapehu	FWVZ	Generalized Only	cvv_WIZ_FWVZ_KRVZ_ONTA_COP_SHW	Multi-volcano (Whakaari, Ruapehu, Tongariro, Ontake, Copahue, St Helens)	Cross-volcano model trained on 5 volcanoes
Augustine	AUH	Generalized Only	cvv_VNSS_BELO_REF_AUH_CETU_GSTR_PVV_OKWR_SHW	Multi-volcano (Veniaminof, Bezymianny, Redoubt, Pavlof, Augustine, Great Sitkin, Semisipochnpo, Okmok, St Helens)	Cross-volcano model trained on 8 volcanoes
Villarrica	VN2Z	Generalized Only	cvv_VN2Z_WIZ_BELO	Multi-volcano	Cross-volcano model (limited eruptions study)
Ontake	ONTA	Generalized Only	cvv_WIZ_FWVZ_KRVZ_ONTA_COP_SHW	Multi-volcano	Cross-volcano model (limited eruptions study)

Supplementary References

1. Newhall, C. G., & Self, S. (1982). The volcanic explosivity index (VEI) an estimate of explosive magnitude for historical volcanism. Journal of Geophysical Research: Oceans, 87(C2), 1231-1238.
2. Yamaoka, K., Geshi, N., Hashimoto, T., Ingebritsen, S. E. & Oikawa, T. Special issue "The phreatic eruption of Mt. Ontake volcano in 2014". Earth Planets Space 68, 175 (2016).

3. Dempsey, D. E., Kempa-Liehr, A. W., Ardid, A., Li, A., Orenia, S. et al. Evaluation of short-term probabilistic eruption forecasting at Whakaari, New Zealand. Bull. Volcanol. 84, 91 (2022).
4. Erfurt-Cooper, P., Sigurdsson, H., & Lopes, R. M. (2015). Volcanoes and tourism. In The encyclopedia of volcanoes (pp. 1295-1311). Academic Press.
5. Auker, M. R., Sparks, R. S. J., Siebert, L., Crosweller, H. S. & Ewert, J. A statistical analysis of the global historical volcanic fatalities record. J. Appl. Volcanol. 2, 2 (2013).
6. Brown, S. K., Jenkins, S. F., Sparks, R. S. J., Odbert, H. & Auker, M. R. Volcanic fatalities database: analysis of volcanic threat with distance and victim classification. J. Appl. Volcanol. 6, 15 (2017).
7. Choumert-Nkolo, J., Lamour, A. & Phelinas, P. The economics of volcanoes. Econ. Disasters Clim. Change 5, 277–299 (2021).
8. Peers, J. B., Gregg, C. E., Lindell, M. K., Pelletier, D., Romerio, F., & Joyner, A. T. (2021). The Economic Effects of Volcanic Alerts—A Case Study of High-Threat US Volcanoes. Risk analysis, 41(10), 1759-1781.
9. Zuccaro, G., Leone, M. F., Del Cogliano, D., & Sgroi, A. (2013). Economic impact of explosive volcanic eruptions: A simulation-based assessment model applied to Campania region volcanoes. Journal of volcanology and geothermal research, 266, 1-15.
10. Bonadonna, C., Asgary, A., Romerio, F., Zulemyan, T., Frischknecht, C., Cristiani, C., & Ricciardi, A. (2022). Assessing the effectiveness and the economic impact of evacuation: the case of the island of Vulcano, Italy. Natural Hazards and Earth System Sciences, 22, 1083–1108.
11. MBIE (2017). Kaikōura: Tourist Travel Behaviour and Recovery Framework. Ministry of Business, Innovation and Employment, New Zealand.
12. Ardid, A., Dempsey, D., Caudron, C., Cronin, S. J., Kennedy, B. et al. Ergodic seismic precursors and transfer learning for short term eruption forecasting at data scarce volcanoes. Nat. Commun. 16, 1758 (2025). https://doi.org/10.1038/s41467-025-56689-x
13. TransportNZ (2020). Social cost of road crashes and injuries. New Zealand Transport Agency.
14. NZH (2021). More than $5 million spent on Whakaari investigation. New Zealand Herald.
15. REU (2024). New Zealand court orders millions in fines over deadly volcanic eruption. Reuters.
16. OECD (various years). Value of statistical life guidance. Organisation for Economic Co-operation and Development.
17. USDOT (2023). Guidance on the value of a statistical life. U.S. Department of Transportation.
18. Bonadonna, C., Frischknecht, C., Menoni, S., Romerio, F., Gregg, C. E., Rosi, M., ... & Cristiani, C. (2021). Integrating hazard, exposure, vulnerability and resilience for risk and emergency management in a volcanic context: the ADVISE model. Journal of applied volcanology, 10(1), 7.
19. Fearnley, C. J., Bird, D. K., Haynes, K., McGuire, W. J., & Jolly, G. (Eds.). (2018). Observing the volcano world: volcano crisis communication. Cham: Springer International Publishing.

20. McDonald, G., Smith, N., Kim, J.-H. & Cronin, S. The spatial and temporal 'cost' of volcanic eruptions: assessing the economic impact, business inoperability, and spatial distribution of risk in the Auckland region, New Zealand. Bull. Volcanol. 79, 48 (2017).
21. McDonald, G., Cronin, S., Kim, J.-H., Smith, N., Murray, C. et al. Computable general equilibrium modelling of economic impacts from volcanic event scenarios at regional and national scale, Mt. Taranaki, New Zealand. Bull. Volcanol. 79, 87 (2017).
22. Marzocchi, W. & Woo, G. Probabilistic eruption forecasting and the call for an evacuation. Geophys. Res. Lett. 34, L22310 (2007).
23. Marzocchi, W. & Bebbington, M. S. Probabilistic eruption forecasting at short and long time scales. Bull. Volcanol. 74, 1777–1805 (2012).
24. Logan, T. M., Aven, T., Guikema, S. & Flage, R. The role of time in risk and risk analysis: implications for resilience, sustainability, and management. Risk Anal. 41, 1959–1970 (2021).
25. Ruapehu District Council (2024). Annual Plan 2023–24.
26. WikiOhakune (2024). Ohakune. Wikipedia.
27. Keys, H. J. (2007). Lahars of Ruapehu volcano, New Zealand: risk mitigation. Annals of Glaciology, 45, 155–162.
28. WikiTangiwai (2024). Tangiwai disaster. Wikipedia.
29. Johnston, D. M., Houghton, B. F., Neall, V. E., Ronan, K. R. & Paton, D. Impacts of the 1945 and 1995-1996 Ruapehu eruptions, New Zealand: an example of increasing societal vulnerability. Geol. Soc. Am. Bull. 112, 720–726 (2000).
30. Fujimi, T., Kono, T., Tatano, H., & Kakimoto, R. (2023). Meta-analysis on value of statistical life in Japan. Japanese Journal of JSCE, 79, n/a. https://doi.org/10.2208/jscejj.23-20008
31. ONEMI (2013). Alerta Roja por actividad del Volcán Copahue. Oficina Nacional de Emergencia, Chile.
32. Prata, F., & Rose, B. (2015). Volcanic ash hazards to aviation. In The Encyclopedia of Volcanoes (pp. 911–934). Academic Press.
33. Belousov, A., Voight, B., & Belousova, M. (2007). Directed blasts and blast-generated pyroclastic density currents: comparison of Bezymianny 1956, Mount St Helens 1980, and Soufrière Hills 1997 eruptions. Bulletin of Volcanology, 69, 701–740.
34. Guffanti, M., Casadevall, T. J., & Budding, K. E. (2010). Encounters of aircraft with volcanic ash clouds: 1953–2009. U.S. Geological Survey Scientific Investigations Report 2010–5117.
