# Supplementary material for: Socio-economic value of data-driven eruption forecasts to balance false alarms against catastrophic loss
Source: Nat Commun. 2026 Aug 26;17:9356. doi: 10.1038/s41467-026-77242-4 (PMC13534426; doi:10.1038/s41467-026-77242-4)
Supplement: Supplementary file 2 [file 41467_2026_77242_MOESM2_ESM.pdf]

nature portfolio

Peer Review File

Socio-economic value of data-driven eruption forecasts to balance false alarms against catastrophic loss

Corresponding Author: Dr Alberto Ardid

This file contains all reviewer reports in order by version, followed by all author rebuttals in order by version.

Version 0:

Reviewer comments:

Reviewer #1

(Remarks to the Author)
This is an interesting, thorough and well written paper that is highly relevant for volcanologists and volcano observatories as they seek to manage the complexity of volcanic risk. It applies a commonly used framework for evaluating the cost-effectiveness of hazard forecasts to the volcanic context. While there is a lot of additional complexity in practice for observatories, this has some potential at the very least as an indicator of the relative significance of missed eruptions over false alarms (a question that poses many challenges in volcano forecasting).
I think that this paper makes a valuable contribution and should be published but I have some suggestions to improve its clarity. It is very clearly building on a previous paper by the authors in this journal and another paper that I could not find either in the references or by a quick literature search (Ardid and Dempsey 2025). The paper could be much clearer in the links it makes with these sources and in defining its own terms. For one thing, the meaning of “decision threshold” (which is an absolutely central concept here) is not clearly defined in the paper in terms of what it means in practice for a user.
The impacts of false alarms are not purely economic (and nor are those of missed eruptions!), and there is some vague recognition of this in the paper, but perhaps scope for more discussion – e.g. of the psychological impacts of repeated false alarms on populations (and on scientists and authorities). Furthermore, there is an underlying assumption here that forecasts automatically lead to action, which is clearly not the case in practice, since this depends upon the provision and uptake of early warnings, which is dependent on good communication and political buy-in (the latter not least in accepting some economic hit for a ‘false alarm’ – and this could be extremely challenging in practice, where political actors come under considerable local pressure from industry – several of the case study volcanoes are in locations where this has been exhibited in the past, especially where the tourist industry has considerable lobbying power).
The paper deals very thoroughly with its cost assumptions and generally does a good job of justifying them (mostly in the SI). It perhaps could be a little clearer about the limitations of the model and its assumptions more broadly – it is a useful tool but it is also a blunt instrument that heavily reduces a complex forecasting exercise. This is evident to some extent in the results, which demonstrate the significant differences between individual volcanoes in terms of populations, costs and monitoring infrastructure. There is a really interesting tension here between the ergodicity in the method and the range of scenarios for individual case study volcanoes, and the authors do a good job of discussing the implications for operational forecasting.
The paper clearly demonstrates the value of this method for volcanology, and particularly in addressing the issue of so-called false alarms. This then requires significant work with communities and civil authorities to enhance their understanding of the limitations of forecasting and the fact that some alerts will result in no eruption. This is actually where the main challenges would arise from this method, and the authors do acknowledge this in the discussion to some extent. There is a lot of information provided in the SI and I think enough to reproduce the results in combination with the references to previous work by the same authors. It is carefully written, and the results do seem to back up the conclusions.
Overall, the paper does a really excellent job of presenting its model and the results, and it does include a number of very useful caveats, limitations and challenges that are inherent to volcano monitoring and forecasting. There are typos throughout that do need addressing. In particular, the use of significant figures is not consistent (especially in figures – e.g. the decision threshold value in 2b), and there are typos also in figure captions (e.g. end of Fig 2 caption ‘five’ not ‘fire’). I think it could be published with minor edits.

(Remarks on code availability)

Reviewer #2

(Remarks to the Author)
1. The primary strength of the paper is the identification of a value-maximizing decision threshold. However, there is a significant "hindsight bias" challenge here. The authors acknowledge that the optimal threshold is only known retrospectively. In a prospective operational setting, how do the authors propose an agency assumption starting threshold? If they choose a threshold that is too risky (i.e. 0.90) and a catastrophic event occurs, the PEV collapses immediately. What can the authors recommend for a Bayesian approach to update the threshold as new data can be made available?
2. Using a statistic "Loss per Missed eruption" might hide the true utility of the authors' forecast. A "missed" eruption during the off-season has a much lower socio-economic cost as comapred to one during the peak tourism season/s. Could the PEV framework be made dynamic, such that it could be that the authors can considered weighting the "L" based on real-time or seasonal exposure data?
3. Does the framework account for compounding cost of false alarms? From what I observe in reality, the cost of the 10th false alarm is higher than the 1st because it degrades the effectiveness of future warnings. Could a variable, say a trust-decay, be considered into the denominator of the PEV formula?
4. Table 1 shows that generalized models outperform tailored models. However, I find this counter-intuitive. It suggest that local expertise/data might actually lead to overfitting or missing broader precursory patterns. Can the authors elaborate further on this? Is it simply a matter of larger training N, or does it suggest that phreatic precursors are more universal than previously thought?
5. How does the PEV framework address political viability? A high PEV score might be satisfy an economist, but if presented to the local tourism board, a loss of US$0.5M a day may send red flags to them. Should the PEV be partitioned into Private sector losses vs Public safety gains?
6. The paper uses VSL benchmarks. Since PEV is a dimensionless ratio, does the choice of VSL significantly shift the location of the optimal threshold or just the magnitude of savings? If the optimal threshold remains stable across different VSLs, that would be a strong argument for the framework's ethical robustness.
7. The study compares ML models against RSAM and finds it is still able to provide positive values. Given that RSAM is easier to implement and explain to the public, is the marginal gain of the ML model high enough to justify the loss in transparency? The authors should address the interpretability vs performance trade-off more explicitly.
8. The references are inconsistent in formatting as well as it is not alphabetized. It is strongly suggested that the authors revise the formatting and presentation of references.

(Remarks on code availability)

Version 1:

Reviewer comments:

Reviewer #1

(Remarks to the Author)
Thank you for the revisions.
I think that the manuscript is now improved with the appropriate clarifications and limitations. It introduces a potentially useful tool that could be applied. It is very reductive of what is a highly complex and place-specific issue, but that does not mean that it has no value and I think the paper is sufficiently clear about its applicability and the trade-off between generalisability and attention to complexity. As a tool, it has potential to be used alongside consideration of place-specific social issues.

Minor points
Should there be an additional subheading for Copahue and Bezimianny in the results section?

(Remarks on code availability)

Reviewer #2

(Remarks to the Author)
The responses completely satisfy the comments and clarifications that I have. The authors did great in responding to each comment that I raised.

(Remarks on code availability)

Open Access This Peer Review File is licensed under a Creative Commons Attribution 4.0 International License, which permits use, sharing, adaptation, distribution and reproduction in any medium or format, as long as you give appropriate credit to the original author(s) and the source, provide a link to the Creative Commons license, and indicate if changes were made.
In cases where reviewers are anonymous, credit should be given to 'Anonymous Referee' and the source.
The images or other third party material in this Peer Review File are included in the article’s Creative Commons license, unless indicated otherwise in a credit line to the material. If material is not included in the article’s Creative Commons license and your intended use is not permitted by statutory regulation or exceeds the permitted use, you will need to obtain permission directly from the copyright holder.
To view a copy of this license, visit https://creativecommons.org/licenses/by/4.0/

Point-by-Point Response to Reviewers

Reviewer comments are shown in **bold**, our responses in plain text, and new or revised manuscript text in *italics*.

Reviewer #1

We thank Reviewer #1 for their positive and constructive assessment. We are pleased that the reviewer finds the paper "interesting, thorough and well written" and recommends publication with minor edits. We address each of their points below.

Definition of decision threshold

"the meaning of 'decision threshold' (which is an absolutely central concept here) is not clearly defined in the paper in terms of what it means in practice for a user."

We agree and have added a clarifying paragraph early in the Results (after the first use of the term) that defines the decision threshold in operational terms.

New manuscript text added to the Results:

In operational terms, the decision threshold τ is the forecast eruption probability above which protective action is initiated: when the model output exceeds τ, the framework recommends precautionary measures such as access restrictions, evacuation of exposed populations, or heightened monitoring posture. A lower τ produces more alerts (including more false alarms), while a higher τ produces fewer alerts but risks missing eruptions preceded by weaker precursory signals.

Ardid and Dempsey (2025) reference

"another paper that I could not find either in the references or by a quick literature search (Ardid and Dempsey 2025)"

This paper has now been published: Ardid, A. et al. Ergodic seismic precursors and transfer learning for short term eruption forecasting at data scarce volcanoes. Nat. Commun. 16, 1758 (2025). https://doi.org/10.1038/s41467-025-56689-x. It is cited as reference 19 in the manuscript.

Non-economic impacts of false alarms and forecast-to-action assumption

"The impacts of false alarms are not purely economic... there is an underlying assumption here that forecasts automatically lead to action, which is clearly not the case in practice, since this depends upon the provision and uptake of early warnings, which is dependent on good communication and political buy-in"

We agree on both counts. We have expanded the trust-decay Discussion paragraph (added for Reviewer #2, Comment 3) to draw on the 'crying wolf' literature from natural hazard warnings, including the tsunami warning analogue that the reviewer alludes to. Tsunami warning systems routinely operate with high false-alarm ratios yet maintain effective evacuation compliance, suggesting that warning credibility depends on risk communication practices and institutional trust rather than alarm frequency alone.

We have also added a new paragraph acknowledging the forecast-to-action assumption explicitly, noting that successful implementation requires institutional willingness, effective communication, and political acceptance of precautionary costs, particularly in settings where tourism operators exert significant lobbying pressure.

New manuscript text added to the Discussion (Operational Integration section):

Future extensions of the PEV framework could incorporate a dynamic trust variable that decays after false alarms and recovers after correct alerts, following the socio-hydrological warning models (Sawada et al., 2022). The 'crying wolf' hypothesis (Dow & Cutter, 1998) predicts that repeated false alarms erode public compliance, and some studies support this for tornado warnings (Simmons & Sutter, 2009). However, others do not replicate the finding for protective behaviour (Trainor et al., 2015; Lim et al., 2019), and tsunami warning systems routinely operate with high false-alarm ratios while maintaining effective evacuation compliance, suggesting that warning credibility depends on risk communication practices and institutional trust rather than alarm frequency alone. Our threshold-and-hold post-processing scheme (Fig. 4) and the recommended reframing of alerts as risk indicators rather than binary predictions provide practical mechanisms for managing alert fatigue without requiring explicit trust modelling.

We acknowledge that this framework assumes that forecasts, once issued, lead to protective action. In practice, the translation of forecast to action depends on institutional decision-making, effective communication, and political acceptance of precautionary costs, particularly where tourism operators exert significant lobbying pressure (e.g., Whakaari, Copahue). The PEV framework supports this translation by providing quantitative evidence that forecast-based action yields net economic benefit, but successful implementation requires engagement with communities and civil authorities to build understanding of forecast uncertainty and acceptance that some alerts will not be followed by eruption.

Reviewer #2

We thank Reviewer #2 for their thorough and constructive engagement with the manuscript. Their comments have improved the clarity and rigour of the paper. Below we address each point in turn. Throughout our responses, we draw on established precedent from fields facing analogous decision problems, including earthquake early warning, flood forecasting, hydrological prediction, and natural hazard risk communication, and discuss how these precedents translate to the volcanic forecasting context.

Comment 1: Hindsight bias / prospective threshold selection

The primary strength of the paper is the identification of a value-maximizing decision threshold. However, there is a significant "hindsight bias" challenge here. The authors acknowledge that the optimal threshold is only known retrospectively. In a prospective operational setting, how do the authors propose an agency assumption starting threshold? If they choose a threshold that is too risky (i.e. 0.90) and a catastrophic event occurs, the PEV collapses immediately. What can the authors recommend for a Bayesian approach to update the threshold as new data can be made available?

We agree that prospective threshold selection is a critical operational challenge, and we thank the reviewer for pushing us to address this more concretely. The revised manuscript now includes a new paragraph in the Discussion that addresses this directly.

Our key arguments are:

First, the sensitivity analyses already presented in the manuscript (Fig. 3) shows that the PEV optimum is broad and relatively flat across a range of intermediate thresholds (approximately tau = 0.65-0.90). This means that an agency does not need to identify the exact optimum to capture most of the forecast's value; being in the right neighbourhood is sufficient.

Second, we now recommend that agencies adopt a robust initial threshold rather than a value-maximising one. This approach has direct precedent in earthquake early warning, where the USGS ShakeAlert system sets alert thresholds below the target damage threshold to bound the probability of missed alerts at a policy-specified ceiling, accepting a measurable increase in false alerts as an insurance premium (Saunders et al., 2022, Earths Future; Minson et al., 2019, Sci. Rep.). We propose an analogous approach for volcanic alerts: begin with a balanced threshold (e.g., tau ~ 0.65), which our sensitivity analysis shows maintains positive PEV across wide cost ranges, and accept the resulting false-alarm rate as the cost of bounding catastrophic-miss probability.

Third, Bayesian updating of the threshold as new forecast-outcome pairs accumulate is well-established in weather and flood forecasting. Martina et al. (2006, HESS) derived prospective flood-warning thresholds by minimising posterior expected loss when both the forecast and loss function are uncertain, with the threshold updated as new data arrive. Katz and Ehrendorfer (2006, Weather Forecast.) showed that Bayesian updating of the predictive distribution gives a sequentially refined cost-loss-optimal threshold. We now propose that the PEV-optimal threshold could be re-estimated periodically (e.g., annually or after each eruptive episode) using a Bayesian posterior over the ROC operating point, and we identify this as a concrete direction for operational implementation.

The reviewer's scenario of choosing tau = 0.90 and then experiencing a catastrophic miss is precisely the outcome our sensitivity analysis warns against (Fig. 3, right panels). Risk-averse thresholds (tau = 0.90) erode value rapidly because even a single missed eruption collapses the economic benefit. This is why we recommend starting at balanced thresholds and adjusting, rather than starting at the retrospective optimum.

New manuscript text added to the Discussion (Operational Challenges section):

In practice, we recommend that agencies adopt a robust initial threshold, one that maintains positive PEV across a broad range of plausible cost assumptions, rather than attempting to identify a value-maximising threshold a priori. Our sensitivity analyses (Fig. 3) indicate that balanced thresholds (tau ~ 0.65-0.70) sustain positive PEV across wide cost-ratio ranges, making them robust initial choices. This approach parallels earthquake early warning practice, where alert thresholds are set below the target damage threshold to bound miss probability at a policy-specified ceiling, accepting a measurable increase in false alerts (Saunders et al., 2022; Minson et al., 2019). As forecast-outcome data accumulate, thresholds can be refined through Bayesian updating of the posterior expected loss, following established methods in flood and weather forecasting (Martina et al., 2006; Katz and Ehrendorfer, 2006). This sequential approach protects against the catastrophic-regret scenario in which a too-permissive threshold is chosen and a major eruption is missed.

Comment 2: Dynamic / seasonal loss weighting

Using a statistic "Loss per Missed eruption" might hide the true utility of the authors' forecast. A "missed" eruption during the off-season has a much lower socio-economic cost as compared to one during the peak tourism season/s. Could the PEV framework be made dynamic, such that it could be that the authors can considered weighting the "L" based on real-time or seasonal exposure data?

This is a well-taken point. We agree that a time-varying loss function would better capture the true distribution of risk and is a natural extension of the framework. The manuscript already notes this heterogeneity (e.g., "exposure at Ruapehu peaks during the winter ski season (~10,000 people per day), while Whakaari hosted ~70 people per day on average"), but we have now added explicit discussion of dynamic loss weighting as a future extension.

We make two important points in response:

First, our current fixed-L approach is conservative with respect to the reviewer's concern. Because L is held constant, the framework does not distinguish between a miss during peak exposure and one during the off-season. A time-varying L(t), weighted by seasonal visitor counts, time-of-day exposure, or permit data, would increase the estimated value of forecasts at high-exposure times, meaning our reported PEV values represent a lower bound on the true forecast value during the periods when it matters most.

Second, dynamic loss weighting has well-established precedent in adjacent hazard domains. Flood-damage assessment now routinely integrates damage over time-varying exposure (Lazzarin et al., 2022, J. Hydrol.), and hurricane evacuation models explicitly condition decision thresholds on population at risk and lead time (Regnier and Harr, 2006, Weather Forecast.). A state-conditioned PEV, with a lower (more precautionary) threshold during peak visitation and a higher threshold off-season, is a natural extension that we identify as future work.

An advantage of the general fixed-L framework is that it can be applied at any volcano with minimal site-specific data requirements. Introducing dynamic L represents a site-specific refinement that could be implemented using even simple proxies such as time of year or time of day, and would strengthen PEV estimates at well-instrumented volcanoes.

New manuscript text added to the Discussion (Model Assumptions and Data Constraints section):

A natural extension of the framework is to replace the scalar missed-eruption loss L with a time-varying function L(t) that reflects seasonal or real-time exposure, following established practice in flood-damage assessment (Lazzarin et al., 2022) and hurricane warning decision models (Regnier and Harr, 2006). At Whakaari, for example, L(t) could be indexed to daily visitor manifests; at Ruapehu, to ski-field attendance. Because our fixed-L approach does not weight misses by exposure, the reported PEV values are conservative: dynamic weighting would increase estimated forecast value during peak-exposure periods when the consequences of a miss are greatest.

Comment 3: Compounding cost of false alarms / trust decay

Does the framework account for compounding cost of false alarms? From what I observe in reality, the cost of the 10th false alarm is higher than the 1st because it degrades the effectiveness of future warnings. Could a variable, say a trust-decay, be considered into the denominator of the PEV formula?

We appreciate this point and agree that trust dynamics are operationally important. However, we offer two responses: one about the empirical evidence, and one about the practical implications for the framework.

First, the empirical evidence for a cumulative false-alarm trust-decay effect is contested in the warning-response literature. The 'crying wolf' hypothesis (Dow & Cutter, 1998, Coast. Manag.) predicts that repeated false alarms erode public compliance, and Simmons and Sutter (2009, Weather Clim. Soc.) found that increasing false-alarm ratios were associated with more tornado casualties, consistent with trust erosion. However, several subsequent studies have not replicated this effect on protective behaviour (Trainor et al., 2015, WCAS; Lim et al., 2019, WCAS). LeClerc and Joslyn (2015, Risk Analysis) found a non-monotonic trust response in controlled experiments. Ripberger et al. (2015, Risk Analysis) showed that perceived (not actual) false-alarm rates predict behaviour, and that both false alarms and missed events erode trust.

Tsunami warning systems provide an instructive analogue: they routinely operate with high false-alarm ratios (the majority of Pacific tsunami warnings do not result in damaging waves) yet maintain sufficient public compliance for effective evacuation. This suggests that warning credibility depends on risk communication practices and institutional trust rather than alarm frequency alone. Given this mixed evidence, we present trust dynamics as a modelling choice requiring calibration rather than a settled empirical law.

Second, our paper already addresses the operational dimension of this concern through two mechanisms: (i) the threshold-and-hold post-processing scheme (Fig. 4), which reduces warning-active time to 7.2% of days at Whakaari while preserving early-warning capability, directly mitigating alert fatigue; and (ii) the Discussion on reframing alerts as indicators of elevated risk rather than binary eruption predictions (analogous to fire-danger

rating systems), which evidence from risk-communication research suggests can sustain credibility even under imperfect forecasts (Doyle et al., 2014).

We have now added acknowledgement of trust-decay modelling as a future extension, presenting some options from the literature. The socio-hydrological framework of Sawada et al. (2022, HESS) provides a direct template, and the 'crying wolf' literature (Dow & Cutter, 1998) provides the foundational hypothesis.

New manuscript text added to the Discussion (Operational Integration and Behavioural Considerations section):

Future extensions of the PEV framework could incorporate a dynamic trust variable that decays after false alarms and recovers after correct alerts, following the socio-hydrological warning model of Sawada et al. (2022). The 'crying wolf' hypothesis (Dow & Cutter, 1998) predicts that repeated false alarms erode public compliance, and some studies support this for tornado warnings (Simmons & Sutter, 2009). However, others do not replicate the finding for protective behaviour (Trainor et al., 2015; Lim et al., 2019), and tsunami warning systems routinely operate with high false-alarm ratios while maintaining effective evacuation compliance, suggesting that warning credibility depends on risk communication practices and institutional trust rather than alarm frequency alone. Our threshold-and-hold post-processing scheme (Fig. 4) and the recommended reframing of alerts as risk indicators rather than binary predictions provide practical mechanisms for managing alert fatigue without requiring explicit trust modelling.

We acknowledge that this framework assumes that forecasts, once issued, lead to protective action. In practice, the translation of forecast to action depends on institutional decision-making, effective communication, and political acceptance of precautionary costs, particularly where tourism operators exert significant lobbying pressure (e.g., Whakaari, Copahue). The PEV framework supports this translation by providing quantitative evidence that forecast-based action yields net economic benefit, but successful implementation requires engagement with communities and civil authorities to build understanding of forecast uncertainty and acceptance that some alerts will not be followed by eruption.

Comment 4: Generalised vs tailored models

Table 1 shows that generalized models outperform tailored models. However, I find this counter-intuitive. It suggests that local expertise/data might actually lead to overfitting or missing broader precursory patterns. Can the authors elaborate further on this? Is it simply a matter of larger training N, or does it suggest that phreatic precursors are more universal than previously thought?

We thank the reviewer for highlighting this point, which we agree deserves fuller explanation. The answer is both: larger effective training N and evidence that seismic precursors share transferable features across volcanoes.

The hypothesised mechanism is that multi-task learning benefits from pooling when per-site sample sizes are small, a pattern now observed across multiple domains. The clearest precedent comes from hydrological prediction in ungauged basins. Kratzert et al. (2019, Water Resour. Res.) trained a single LSTM on 531 catchments and showed that this regional model outperformed individually calibrated conceptual models in each basin, even at gauged sites. The pooled network learns process invariances (shared rainfall-runoff dynamics) that

small per-site samples cannot resolve, while per-site models overfit to local noise. Nearing et al. (2021) and Arsenault et al. (2023) replicated this result, and it is now considered a general regularity in hydrological ML.

For the volcanic application specifically, the generalized model's advantage draws directly from the finding of Ardid and Dempsey et al. (2025, ref. 19) that certain seismic precursory features, particularly in the spectral and amplitude domains, transfer across volcanic systems with different magmatic compositions and eruption styles. This supports the interpretation that phreatic/seismic precursors are more universal than previously assumed, at least at the level of the features extracted by the ML pipeline. The tailored model, trained on only 5-6 eruptions at a single volcano, is susceptible to overfitting to volcano-specific noise patterns and to missing precursory features that are shared across systems but weakly expressed at any individual site.

We note that this pattern is not universal across all volcanoes in our dataset: at Bezymianny, for example, the tailored model's PEV is competitive with the generalised model (Table 1). However, the overall trend strongly favours generalised training in the data-scarce regime.

We have expanded the relevant Discussion text to make this argument explicit.

New manuscript text added to the Discussion (new paragraph after model comparison section):

The finding that generalized models outperform tailored models (Table 1) is consistent with a well-established regularity in multi-task machine learning applied to data-scarce settings. In hydrological prediction, Kratzert et al. (2019) showed that a single LSTM trained across 531 catchments outperformed individually calibrated models at each site, because the pooled network captures process invariances that small per-site samples cannot resolve. With 5-6 eruptions per volcano (Table 1), our dataset is in the regime where pooled training has been shown to outperform per-site calibration in other domains. This result also supports the interpretation that seismic precursory features are more universal across volcanic systems than previously assumed, consistent with the transfer-learning findings of Ardid and Dempsey et al. (2025).

Comment 5: Political viability / stakeholder partitioning

How does the PEV framework address political viability? A high PEV score might satisfy an economist, but if presented to the local tourism board, a loss of US$0.5M a day may send red flags to them. Should the PEV be partitioned into Private sector losses vs Public safety gains?

We agree that the distributional dimension of forecast costs is important for policy acceptance, and we note that our manuscript already addresses this directly. The section "Distribution of forecast-related costs and societal acceptance" (p. 11) discusses the asymmetry between who bears false-alarm costs (tourism operators, visitors) and who benefits from avoided catastrophic losses (exposed individuals, public agencies). Supplementary Text S3 and Figure S3 provide a hypothetical analysis of this cost-incidence asymmetry.

The reviewer's suggestion of a stakeholder-partitioned PEV is well-aligned with established practice in regulatory cost-benefit analysis, where distributional analysis decomposes aggregate metrics by affected subgroup. This approach is standard in US EPA environmental justice regulatory analysis (Robinson et al., 2016, Rev. Environ. Econ. Policy).

We have added text to the Discussion making clear that PEV quantifies the aggregate economic value added by the forecast, and that the stakeholder decomposition (already presented in Supplementary Text S3) informs how the resulting costs and benefits are distributed across affected groups. We now reference distributional cost-benefit analysis as a formal framework for this decomposition.

However, we note that fully partitioning PEV by stakeholder group would require detailed, site-specific data on cost incidence that is not uniformly available and would reduce the framework's generalizability, which is a core contribution of the paper. We frame stakeholder partitioning as a site-specific refinement to be applied when PEV is used in local policy deliberation.

New manuscript text added to the Discussion (Distribution of forecast-related costs section):

We emphasise that PEV is intended as a decision-support tool for facilitating transparent stakeholder deliberation, not as a prescriptive metric that determines policy. A stakeholder-partitioned analysis, decomposing aggregate PEV into private-sector costs (e.g., tourism revenue losses from closures) and public-safety gains (e.g., avoided fatalities and emergency response costs), can inform how the costs and benefits of precautionary action are distributed across affected groups. Supplementary Text S3 provides an illustrative decomposition for Whakaari. Formal methods for such distributional analysis are well-established in environmental regulatory analysis (Robinson et al., 2016), and represent a natural extension of the PEV framework for site-specific policy application.

Comment 6: VSL sensitivity on threshold location

The paper uses VSL benchmarks. Since PEV is a dimensionless ratio, does the choice of VSL significantly shift the location of the optimal threshold or just the magnitude of savings? If the optimal threshold remains stable across different VSLs, that would be a strong argument for the framework's ethical robustness.

The reviewer raises an important question about the sensitivity of the framework to the choice of VSL.

The key insight is that PEV-optimal thresholds depend on the cost-loss ratio C/L, not on the absolute magnitudes of C and L individually. This is a well-known property of cost-loss decision frameworks (Richardson, 2000, QJRMS; 2012, Forecast Verification). Since PEV is defined as:

PEV(tau) = 1 - [(C/L) * b(tau) + m(tau)]

the optimal threshold is determined by C/L together with the forecast's ROC performance.

The false-alarm cost C is primarily composed of fixed operational expenditures: evacuation logistics, lost tourism revenue per closure day, and emergency service mobilization. These costs are largely independent of how one values a statistical life. By contrast, the missed-eruption loss L includes VSL multiplied by expected fatalities as a major component, and therefore scales with the chosen VSL benchmark.

This means that increasing VSL increases L while leaving C largely unchanged, which decreases C/L and shifts the optimal threshold toward lower, more precautionary values. Importantly, this directional effect actually

strengthens the case for precautionary alerting: a higher valuation of human life lowers the bar for protective action within the PEV framework, consistent with ethical intuition.

The framework's practical conclusions are robust for two reasons. First, the sensitivity analyses in Fig. 3 show that PEV remains positive across a wide range of C/L values, so even substantial changes in VSL (and hence C/L) do not change the qualitative conclusion that forecasts add economic value. Second, the PEV curve is broad and flat around its optimum, meaning that the shift in optimal threshold location across plausible VSL ranges is small relative to the range of thresholds over which PEV is positive. The framework's policy-relevant conclusions, namely which thresholds provide positive value and that balanced thresholds are preferred, are therefore robust to the choice of VSL.

New manuscript text added to the Discussion (after existing VSL paragraph):

The false-alarm cost C is primarily composed of fixed operational expenditures (evacuation logistics, lost revenue, emergency service mobilization) that are largely independent of VSL, whereas the missed-eruption loss L scales with the chosen VSL benchmark through its fatality component. Increasing VSL therefore decreases C/L and shifts the optimal threshold toward more precautionary values, consistent with the ethical intuition that greater weight on human life should lower the bar for protective action. The sensitivity analyses in Fig. 3 show that PEV remains positive across a wide range of C/L values, and the broad, flat optimum means that shifts in optimal threshold location across plausible VSL ranges are small. The framework's policy-relevant conclusions, namely which thresholds provide positive value and that balanced thresholds are preferred, are therefore robust to the choice of VSL benchmark.

Comment 7: Interpretability vs performance (RSAM vs ML)

The study compares ML models against RSAM and finds it is still able to provide positive values. Given that RSAM is easier to implement and explain to the public, is the marginal gain of the ML model high enough to justify the loss in transparency? The authors should address the interpretability vs performance trade-off more explicitly.

This is a fair and important point that we have now addressed more explicitly in the manuscript.

We make three arguments:

First, RSAM on its own makes the PEV case. Our results show that RSAM provides positive PEV at all three volcanoes where it was tested (Table 1), confirming that even the simplest, most transparent monitoring metric has demonstrable forecast value within the PEV framework. This is an important result: agencies with limited technical capacity can achieve positive economic value from forecast-based action using RSAM alone, with no ML infrastructure required.

Second, the interpretability concern raised by Rudin (2019, Nat. Mach. Intell.), that inherently interpretable models should be preferred in high-stakes decisions unless the accuracy gain from opaque models is substantial, applies here. We take this concern seriously.

Third, the marginal gain from ML is nonetheless meaningful in absolute terms. Across the three volcanoes in Table 1, the generalized ML model provides USD 30-115M in additional savings over RSAM (comparing generalized model savings to RSAM savings: Whakaari +114M, Bezymianny +1M, Copahue +35M). This gain arises because ML models detect subtler precursory patterns that RSAM, a simple amplitude metric, cannot resolve. We note that the ML models used here are partially interpretable through feature importances and partial-dependence plots, and the underlying features are derived from physically meaningful seismic properties. The models are therefore not "black boxes" in the sense of deep neural networks with opaque internal representations.

We now propose in the Discussion that RSAM and ML models can serve complementary roles: RSAM as a transparent baseline that agencies and the public can understand, and ML as a supplementary decision-support layer that provides quantifiable additional value. The choice between them is not binary but depends on local institutional capacity, data infrastructure, and communication needs.

New manuscript text added to the Discussion (new paragraph after model comparison section):

An important operational consideration is the trade-off between forecast performance and interpretability. RSAM, as a simple seismic amplitude metric, is straightforward to implement, explain to stakeholders, and audit, and our analysis confirms that it provides positive economic value at all tested volcanoes (Table 1). The ML models provide additional value (USD 30-115M in marginal savings over RSAM across the tested volcanoes) by detecting subtler precursory patterns, and are partially interpretable through feature importances and physically meaningful input features. In operational settings, RSAM and ML models need not be competing alternatives: RSAM can serve as a transparent baseline that agencies and the public can readily understand, while ML outputs provide supplementary decision support with quantifiable additional value (Rudin, 2019). The choice of which to deploy depends on local institutional capacity, data infrastructure, and the communication context in which forecasts are used.
